# Towards precision EEG connectomics: Evaluating the benefits of dense sampling

**DOI:** 10.1162/IMAG.a.1245

**Published:** 2026-06-04

**Authors:** Kirk Graff, Shefali Rai, Shelly Yin, Kate J. Godfrey, Daria Merrikh, Ryann Tansey, Tamara Vanderwal, Andrea B. Protzner, Signe Bray

**Affiliations:** Child and Adolescent Imaging Research Program, University of Calgary, Calgary, AB, Canada; Alberta Children’s Hospital Research Institute, University of Calgary, Calgary, AB, Canada; Hotchkiss Brain Institute, University of Calgary, Calgary, AB, Canada; Department of Community Health Sciences, University of Calgary, Calgary, AB, Canada; Department of Clinical Neurosciences, University of Calgary, Calgary, AB, Canada; Department of Psychiatry, University of Calgary, Calgary, AB, Canada; Mathison Centre for Mental Health Research and Education, University of Calgary, Calgary, AB, Canada; Department of Psychiatry, University of British Columbia, Vancouver, BC, Canada; BC Children’s Hospital Research Institute, Vancouver, BC, Canada; Department of Psychology, University of Calgary, Calgary, AB, Canada; Department of Radiology, University of Calgary, Calgary, AB, Canada

**Keywords:** EEG, functional connectivity, functional connectome, reliability, validity, volume conduction

## Abstract

EEG connectomics research offers the potential to better understand human neurodevelopment, brain disorders, and brain–behavior associations. Several functional connectivity (FC) measures are widely used but few studies have directly compared metrics of reliability across measures and how data quantity influences these properties. Here, we collected a densely sampled dataset from 25 parent–child pairs, with 80 minutes of passive viewing EEG data collected per participant over 4 sessions, and calculated connectomes using 9 popular phase- and envelope-based measures (coherence, COH; phase locking value, PLV; corrected imaginary phase locking value, CIPLV; imaginary coherence, IMCOH; phase-lag index, PLI; weighted phase-lag index, WPLI; envelope correlation with pairwise orthogonalization, ECPWO; and envelope correlation with symmetric orthogonalization, ECSO), including one effective connectivity measure (phase slope index; PSI). We used connectome individualization, derived from fingerprinting-style analyses, as a multivariate reliability metric, as used in fMRI-FC studies. We used simulations with individual head geometry but no “true” connectivity to assess whether identifiability was influenced by volume conduction. We found that COH and PLV were vulnerable to volume conduction influence on identifiability; CIPLV, ECSO, and ECPWO were semi-vulnerable; and IMCOH, WPLI, PLI, and PSI were minimally vulnerable. Next, we considered individualization and reliability of age-group effects with increasing time of data collection. IMCOH had the overall best performance among minimally vulnerable measures, considering individualization and reliability of group effects. We further found that reliability of IMCOH along with other volume conduction-corrected EEG-FC measures continued to improve with up to 25–30 minutes of data. Together, our findings can support study design decisions in EEG connectomics research.

## 1 Introduction

Electroencephalography (EEG) connectomics is a fast-growing subfield of EEG neuroscience research (Sadaghiani et al., 2022). While functional magnetic resonance imaging (fMRI)-based modalities have dominated the connectome literature to date, EEG has the advantages of more directly measuring neural activity, millisecond temporal resolution, and greater cost-effectiveness (Sadaghiani et al., 2022), providing the potential to contribute to a better understanding of human neurodevelopment, disorders, and disease. As EEG biomarkers for different neurological conditions are being actively investigated (Benbadis et al., 2020; Espenhahn et al., 2021; Houmani et al., 2018; Sadaghiani et al., 2019), a growing number of studies are considering the potential for EEG functional connectivity (FC) to provide reliable individual measurements (Hatlestad-Hall et al., 2023; Marquetand et al., 2019; Nagy et al., 2024; Nentwich et al., 2020; Popov et al., 2023; van der Velde et al., 2019). Here, we leverage a unique dataset with repeated EEG recordings to assess reliability and validity considerations for different EEG FC measures with a specific focus on the impact of temporal duration on EEG connectome reliability.

While a relatively small number of functional connectivity metrics (i.e., Pearson correlation, ICA) account for the majority of fMRI research studies, the high temporal resolution and inherent confounds of EEG, including volume conduction and spatial leakage, have led to dozens of EEG FC measures being developed (Cao et al., 2022; Sadaghiani et al., 2022). Here, we focus on phase-based measures and envelope correlations as they are commonly used (Cao et al., 2022; Sadaghiani et al., 2022) and have several advantages, including requiring few assumptions (Cohen, 2014), and capturing distinct information from what is captured by fMRI (Engel et al., 2013; Wirsich et al., 2021).

Measurement reliability, the extent to which multiple recordings from the same individual produce consistent estimates under similar conditions (Noble et al., 2019), can be assessed in a multivariate context by correlating connectomes from different recordings, often referred to as connectome stability (Finn et al., 2015; Valizadeh et al., 2019; Wu et al., 2022). An extension to this approach that considers individual distinctiveness is connectome “fingerprinting” or related identifiability metrics, which measure the gap between self-to-self stability and similarity to others (Finn et al., 2015; Graff et al., 2022; Kaufmann et al., 2017; Rajapandian et al., 2020; Ramduny & Kelly, 2025). High identifiability is generally a desirable property for connectomes, as this implies stable differences between individuals (Graff et al., 2022; Ramduny and Kelly, 2025).

While maximizing reliability is important, this can sometimes come at a cost of reducing validity (Noble et al., 2021). One of the known issues with some EEG measures is volume conduction artifacts that can lead to spurious “connectivity” by measuring the same signal at multiple sources. Generally, studies that have compared the reliability of EEG and/or magnetoencephalography (MEG) FC measures have found that measures that do not include correction for volume conduction have higher reliability (Colclough et al., 2016) and it has been suggested that these measures are, therefore, well suited to biometric identifiability (Fraschini et al., 2019; Tian et al., 2022; Wang et al., 2020). However, simulations show that increased reliability likely comes at the expense of reduced validity in terms of measuring the construct of interest, “true” connectivity that reflects brain activity (Yu, 2020). To examine this trade-off, we use simulations with no “true” underlying connectivity, which allows us to determine the sensitivity of EEG measures to volume conduction effects. As volume conduction artifacts are spatially dependent, a related concern is the extent to which shorter connections may be artificially inflated relative to longer connections (Thatcher et al., 1986). Measures with prominent distance effects may be considered less valid relative to those with reduced distance dependence of connectivity.

While it has been suggested that larger number of epochs are better for reliability (Marquetand et al., 2019), no studies to our knowledge report on reliability and validity using relatively long scans, which are important for identifying the duration at which reliability measures reach an asymptote. This is in contrast to a growing field of precision fMRI connectome research showing an asymptotic relationship between scan duration and connectome reliability (Gordon et al., 2017; Poldrack et al., 2015). Here, we consider how scan duration affects the reliability of connectomes and group differences measured for different metrics.

We leverage a unique dataset in which we collected data from adults and children across four sessions and three passive viewing tasks, for a total of ~80 minutes of EEG data per person. We consider nine commonly used EEG measures of connectivity, including measures sensitive to phase synchrony (phase lag index (PLI; Stam et al., 2007), weighted phase lag index (WPLI; Vinck et al., 2011), phase locking value (PLV; Lachaux et al., 1999), and corrected imaginary phase locking value (CIPLV; Bruña et al., 2018)), measures that are also sensitive to amplitude (coherence (COH; Cohen, 2014) and imaginary coherence (IMCOH; Nolte et al., 2004)), the phase slope index (PSI; Nolte et al., 2008), which estimates causal influences in synchrony (i.e., effective connectivity) and envelope correlations (Hipp et al., 2012), considering two different artifact-correction strategies—symmetric orthogonalization (ECSO; Colclough et al., 2015) and pairwise orthogonalization (ECPWO; Hipp et al., 2012). We began by asking whether, for each measure, volume conduction artificially inflates multivariate reliability. We found that measures had strong (COH, PLV), weak-to-moderate (ECSO, CIPLV, ECPWO), and no (IMCOH, PSI, PLI, WPLI) dependence on head geometry, which inflates reliability at the expense of validity. We then consider these groups of measures separately for the purposes of assessing reliability of connectomes and age-group effects as a function of scan duration. Our findings can support data collection and processing decisions towards reliable individual measurement in EEG connectomics.

## 2 Methods

### 2.1 Participants

Participants were recruited into a study using precision functional mapping to compare brain networks between adults and pre-adolescent children. Given the relatively high burden of this study with repeated visits, we recruited a parent as an adult “control” for each child. The non-independence in the data introduced by family is accounted for in analyses, as described below. We recruited 50 participants, consisting of 25 parent–child dyads (5 female–female, 7 female–male, 8 male–female, 5 male–male). Parents were 33.75–47.13 years old (mean: 41.39, std: 3.63 years, 12 female). Children were 6.56–8.92 years old at the time of first visit (mean: 7.88, std: 0.69 years, 13 female). Participants were recruited from the community, generally healthy, free from MRI contraindications, and did not have a history of seizures or major neurological or psychiatric illness. Our sample included two families where both parents and two children participated. See Supplemental Table S1 for more detailed participant information. Parents provided informed consent for both their own and their child’s participation, while children provided assent. This study was approved by the University of Calgary Conjoint Health Research Ethics Board.

### 2.2 Data collection

Data were collected at the Alberta Children’s Hospital across four sessions per parent–child pair, with each session completed at least 3 days after the previous one (median = 15 days between visits; see Supplemental Table S1). Families completed all four visits within 6 months, with one outlier (467 days from visits 1 to 4). EEG data were collected using a 64-channel Magstim EGI HydroCel Geodesic Sensor Net (Eugene, Oregon), soaked in an electrolytic solution, with data sampled at 1000 Hz. The impedance level was kept below 50 kΩ during recording. MRI data, used for source reconstruction, were collected using a 3T GE MR750 w (Waukesha, WI) scanner with a 32-channel head coil, which consisted of a T1w 3D BRAVO sequence (TR = 6.764 ms, TE = 2.908 ms, FA = 10°, voxel size = 0.8 × 0.8 × 0.8 mm). Functional and diffusion MRI, and other cognitive and behavioral data were also collected but not analyzed for the present study. The order of data collection was pseudo-randomized, that is, for half the participants, parents underwent EEG then MRI data collection, while children underwent MRI then EEG data collection. In the other half, the order was reversed.

Each of the four EEG recording sessions included 3 approximately 7-minute passive video viewing tasks, for a total of 12 unique videos that were drawn from 3 categories:

- A continuous relaxing video with gentle music, slow-moving imagery, and no narration, designed to minimize cognitive load, similar in concept to *Inscapes* (Vanderwal et al., 2015). All participants watched the same videos in the same order across sessions; the videos shown depicted (1) a Japanese island, (2) a walk through a canyon, (3) scenes from Canadian cities, and (4) the Earth as seen from space.
- A series of popular (i.e., viewed more than 1 million times at the time they were downloaded) and visually engaging non-narrative clips accessed on youtube.com (24–65 seconds long, mean = 46.4 seconds), designed to provide constant visual interest with minimal narrative. Clips included dancing, arts and crafts, simple science experiments, scenes from video games, stop-motion animation, and humorous short videos. All participants watched these clips in the same order across sessions.
- A series of scenes from *Dora and the Lost City of Gold* (2019), a live-action movie. This condition was designed to contain a continuous narrative. Over the course of the study, participants viewed successive clips of this movie sequentially during MR and EEG imaging. Whether participants underwent EEG or MRI first determined whether they watched the first sequential video or the last sequential video during EEG recording, that is, half the participants watched clips 1, 3, 6, and 9 during EEG and half watched clips 3, 6, 9, and 12 during EEG.

The EEG recording was timed to begin and end with video playback. Electrodes were checked in between videos to ensure impedances remained below 50 kΩ. Within each session, the video category order was randomized across participants.

In total, 598 EEG recordings were collected in total. Four sessions of data (three recordings each) were excluded due to concerns with correct cap placement and five recordings were excluded due to too many noisy epochs (see section 2.3). This left 581 recordings in the final sample across 50 participants. All participants had at least 8 recordings, with at least 2 recordings of each video type; 42 participants had all 12 recordings.

### 2.3 Preprocessing

Data were preprocessed using MNE-Python (Gramfort et al., 2013). Data were downsampled to 250 Hz, bandpass filtered from 1–45 Hz, and notch filtered at 60 Hz. Channels were then visually inspected to exclude noisy channels (e.g., excessive artifacts or long stretches of no signal). Electrodes were re-referenced to the average signal of all (remaining) electrodes, data were divided into 2-second (non-overlapping) epochs, and an independent component analysis was carried out for each recording to remove eye and other artifacts. Components were classified as brain activity or artifact using mne-icalabel (Li et al., 2022), and classification was confirmed using visual inspection, erring on the side of being conservative with component removal. On average, 4.93 independent components were classified as artifact per recording (range: 2–12, std: 1.67). Following this, each epoch for each channel was checked for noise, where an amplitude exceeding ±70 μV or a change of 100 μV over 100 ms was classified as noisy (Schubert et al., 2015). Channels with noisy epochs were interpolated using data from other channels via spherical spline interpolation. Any epoch with more than 16 unusable channels—either requiring interpolation or having been excluded during visual inspection—was removed from analysis. Recordings with fewer than 150 non-noisy epochs were excluded. While there is some evidence that longer epoch lengths can be advantageous (Fraschini et al., 2016; Nagy et al., 2024), we used shorter epochs to minimize the amount of data needing interpolation or exclusion while minimizing the impact of artifacts.

T1w images were preprocessed using FreeSurfer (Dale et al., 1999). Electrode placements were estimated based on the standard montage for a 64-channel EGI HydroCel Geodesic Sensor Net and by using an automated approach to coregistration with the participant’s MRI (Houck and Claus, 2020). Source localization was carried out using the eLORETA algorithm (Pascual-Marqui, 2007). Source space was defined using six recursively subdivided octahedrons, the MNE-Python default, and the forward solution assumed conductivities of 0.3 S/m, 0.006 S/m, and 0.3 S/m, also default settings, for the scalp, skull, and brain, respectively. For each of the 68 nodes in the Desikan–Killiany atlas (Desikan et al., 2006), the time course was extracted using the first principal component of the vertices via the pca_flip setting.

### 2.4 Simulated brain activity

To test the effects of volume conduction on FC, we simulated EEG data where the underlying brain activity was unstructured noise, using the MNE Source Simulator class. Following the previously described preprocessing, for each EEG recording, we created a new time course in source space for each node in the Desikan–Killiany atlas. This was done by adding together a series of sine waves with random amplitudes and phases, with frequencies ranging from 1 Hz to 60 Hz in 0.1 Hz intervals. This brain activity was then projected into sensor space using the forward solution, with additional Gaussian noise added after this step. These new sensor space data were projected back into source space and parcellated using the same procedure as with the real EEG data. For each real EEG recording, we generated 100 simulated recordings, each 20 seconds long, for a total of 1000 simulated epochs.

Using the same forward solution to both project simulated time courses from source space to sensor space and to project them back to source space could bias results through the “inverse crime” (Kaipio & Somersalo, 2007). We repeated this simulation process for eight participants (four adults and four children) where we used an alternative forward solution to move from source space to sensor space where source space was defined using five recursively subdivided icosahedrons. We also assumed conductivities of 0.332 S/m, 0.0113 S/m, and 0.332 S/m for the scalp, skull, and brain, respectively, consistent with the approach used by Morik et al. (2025). Other details were kept the same, including using the original forward solution to return from sensor to source space. Noise was not reused between the two approaches, so the correlation between the connectomes should be near zero.

### 2.5 Connectivity measures and connectome generation

For both real and simulated EEG data, we calculated nine different connectivity measures, which are summarized in Table 1. All were calculated in MNE-Python. The first seven are phase-based connectivity measures, using multitaper spectrum estimation with adaptive weights, where tapered spectra are combined into power spectral densities. The last two are both envelope correlations, varying in orthogonalization strategy.

**Table 1.** A comparison of FC measure properties considered in this study.

	Sensitive to			
Measure	Phase	Amplitude	Volume conduction mitigation	Effective connectivity
Coherence (COH)	✓	✓		
Phase locking value (PLV)	✓			
Phase lag index (PLI)	✓		✓	
Weighted phase lag index (WPLI)	✓		✓	
Imaginary coherence (IMCOH)	✓	✓	✓	
Corrected imaginary phase locking value (CIPLV)	✓		✓	
Phase slope index (PSI)	✓		✓	✓
Envelope correlation with pairwise orthogonalization (ECPWO)		✓	✓	
Envelope correlation with symmetric orthogonalization (ECSO)		✓	✓

1. The magnitude of coherency, often referred to as coherence (COH; Cohen, 2014; Nolte et al., 2004), is one of the most established techniques to estimate electrophysiological synchrony. It measures the linear covariance between two spectra, incorporating both phase and power information. It does not incorporate any strategy to mitigate volume conduction.
2. Phase locking value (PLV; Lachaux et al., 1999) measures the degree of phase covariance. It was proposed as an alternative to COH that would be effective for non-stationary sources and would only account for phase information, ignoring amplitude covariance. Like COH, it does not incorporate any strategy to mitigate volume conduction.
3. Imaginary coherence (IMCOH; Nolte et al., 2004) involves selecting only the imaginary component from a coherency calculation between two regions. It was proposed as an alternative to COH that mitigates volume conduction artifacts by being insensitive to zero time-lag synchrony between nodes, thereby removing spurious connectivity (while also likely removing true signal of interest). While IMCOH and other zero time-lag synchrony measures avoid most of the direct effects of volume conduction, they are still affected by indirect effects such as field spread artifacts (Palva et al., 2018).
4. Corrected imaginary phase locking value (CIPLV; Bruña et al., 2018) is—like IMCOH with respect to COH—the imaginary part of PLV, normalized to better estimate the upper bound of possible connectivity. In addition to being faster to calculate than the original definition of PLV, this measure is designed to minimize but not completely eliminate the effects of volume conduction, as the authors argue that complete insensitivity to volume conduction can underestimate true connectivity (Bruña et al., 2018).
5. Phase lag index (PLI; Stam et al., 2007) measures the consistency of phase lag between two time series. Like IMCOH, it ignores zero time-lag synchrony—spurious or real—but unlike IMCOH, it is only sensitive to phase covariance, ignoring amplitude effects.
6. Weighted phase lag index (WPLI; Vinck et al., 2011) is a modification of PLI where phase differences are weighted according to the size of the difference, rather than assessing only the consistency of phase difference. It has been shown to be more robust against noise with increased statistical power (Vinck et al., 2011).
7. Phase slope index (PSI; Nolte et al., 2008) is based on the assumption that for an interaction with a given time delay, the phase difference between signals will increase with frequency. It measures the slope of these phase differences, where the sign of the slope indicates which region is responsible for the interaction, making it an effective connectivity measure. Like IMCOH, PLI, and WPLI, it is less affected by volume conduction compared with COH and PLV.
8. Envelope correlation with pairwise orthogonalization (ECPWO; Hipp et al., 2012) assesses the similarity in signal amplitude. To correct for volume conduction, between each pair of signals the components that share the same phase are removed prior to calculating each signal’s power envelope and then the corresponding correlation.
9. Envelope correlation with symmetric, multivariate orthogonalization (ECSO; Colclough et al., 2015) is conceptually similar to ECPWO, but instead corrects the time series for all regions of interest prior to calculating envelope correlations. This is performed by removing correlations with zero temporal lag between all time courses. Note that our procedure differs from Colclough et al. (2015) by carrying out symmetric orthogonalization on individual epochs, similar to Khan et al. (2018).

FC was calculated for broadband (2.5–45 Hz) and two narrower frequency bands, alpha (8–13 Hz) and beta (13–30 Hz) band. We considered alpha and beta bands (rather than, e.g., delta or theta) because some evidence suggests reliability is highest in these bands (Lopez et al., 2023), which may allow validity to be better assessed. FC was calculated between all pairs of regions in the Desikan–Killiany atlas (n = 68), yielding 2278 edges, which were Fisher z-transformed to approximate a normal distribution and vectorized.

### 2.6 Assessing validity through similarity between real and simulated connectomes and distance dependence

For each EEG recording, we calculated the Spearman correlation between the vectorized real EEG connectome and the average of its 100 associated simulated connectomes. Similarly, we calculated the correlation between each (real) connectome and the length of each edge, calculated as the Euclidian distance between the centers of nodes for edge length, using individual-specific coordinates. We also calculated—for both real and simulated connectomes—an average connectome across all 581 recordings and calculated the real-to-simulated correlation and real-to-edge-distance correlation for the averaged connectomes. For IMCOH and PSI, which have both positive and negative values, we used the absolute value of connectivity for calculating distance dependence. We visualized the averaged connectomes as connectivity matrices, with regions divided into hemispheres and arranged anatomically from posterior to anterior. These correlations and visualizations were done for each connectivity measure and frequency band.

### 2.7 Connectivity stability and identifiability

While there are multiple ways to assess connectome reliability, a straightforward approach is to vectorize connectomes and calculate the within-subject test–retest correlation (Colclough et al., 2016; Finn et al., 2015; Gordon et al., 2017), hereafter referred to as self-stability. For each connectivity measure and frequency band, pairs of connectomes from the same participant were Pearson correlated to generate a self-stability score. Correlations were Fisher-z transformed. We calculated between-session self-stability scores to minimize spuriously higher self-stability due to more similar recording conditions, for example, cap placement, other electric fields, and electrolytic solution concentration. Participants had between 23 and 54 (mean = 50.6) between-session self-stability scores. We repeated this procedure with the simulated connectomes, assuming that connectivity measures insensitive to volume conduction or other potential anatomical effects would show no self-stability.

We also considered participant identifiability. For each participant, identifiability was assessed with the difference between average self-stability and average similarity-to-others (Graff et al., 2022). Similarity-to-others was assessed along the same lines as self-stability (Vanderwal et al., 2021), where we calculated the Pearson correlation between pairs of connectomes from different unrelated participants (i.e., not to their parent or their child). For the families where two siblings and both parents participated, siblings were not compared with each other as they are related, but parents were compared with each other (biologically unrelated). We excluded biologically related participants from comparison to avoid potentially higher similarity-to-other scores based on heritability, as this has been previously observed (Miranda-Dominguez et al., 2018). Participants had between 4488 (8 × 561) and 6684 (12 × 557) similarity-to-other scores (mean = 6445). These were likewise Fisher z-transformed.

We further tested two supplemental metrics of individualization:

- Matching accuracy: for each participant, the fraction of self-stability scores that were higher than all similarity-to-others scores.
- Self-stability percentile rank: for each participant, the average percentile of self-stability scores, relative to similarity-to-others. A score of 50% suggests no difference between self-stability and the median similarity to someone else.

Participant identifiability was also calculated for the simulated connectomes, again assuming that individualization should be zero if not for spatial artifacts.

fMRI connectome studies have shown that as recording length increases, reliability increases (Finn et al., 2015; Gordon et al., 2017; Noble et al., 2019). Here, we investigated the extent to which reliability is sensitive to the amount of data available across FC measures. Of the 42 participants with all 12 usable recordings, we concatenated the 6 recordings from their first 2 sessions, and the 6 from their last 2 sessions, generating 2 sets of data. We then calculated self-stability, similarity-to-others, and participant identifiability using progressively larger quantities of data, starting with 1 epoch from each recording (6 in total per set) and increasing up to 6 × 150 epochs per set (30 minutes of data per set, 60 minutes in total).

### 2.8 Reliability of age-group effects comparing children and adults

We explored the reliability of differences due to maturation by comparing the child group with the parent group and assessing whether measured differences were similar across sessions. For the 18 parent–child pairs with all usable recordings, we concatenated recordings from the first 2 sessions and last 2 sessions, generating 2 sets of data. Due to the non-identical ordering of clips from *Dora and the Lost City of Gold,* for each participant, five total recordings were used to generate the first half connectome and five for the second half (rather than six for each, as in the identifiability analysis). This was due to only one *Dora* clip (#3) being watched by both parent and child across the first two sessions; likewise for clip #9 during the last two sessions. We then again used progressively longer sets of data to calculate age-group effects, starting with 1 epoch from each recording, and increasing up to 5 × 150 epochs. For each data quantity and for each of the 2278 edges, we used a paired t-test, pairing parent and child, to assess the difference between adult and child groups. This was done for both sets of data independently, giving a set of T values from the first two sessions and a set of T values from the last two sessions. We then calculated the reliability of age-group effects as the Pearson correlation between these sets of 2278 T values.

## 3 Results

Broadly speaking, results were comparable across frequency bands. However, volume conduction artifacts were more pronounced for broadband than for either alpha or beta band. Given the importance of describing which connectivity measures are sensitive to artifact, here we focus our descriptions on and share figures for broadband connectivity, though note the main differences between the bands where applicable. Figures for alpha band and beta band are available in Supplementary Material.

### 3.1 Real to simulated correlations

**Fig. 1.**
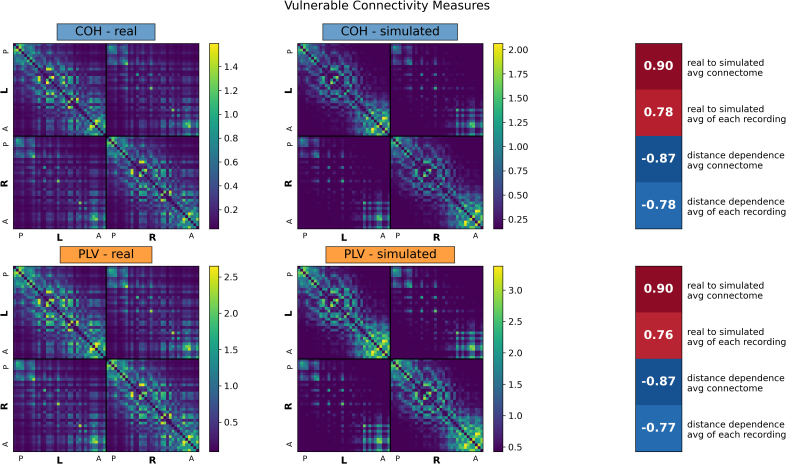
Average real and simulated broadband (2.5–45.0 Hz) connectomes for connectivity measures vulnerable to volume conduction. Averages are based off of all 581 recordings across 50 participants for both real and simulated connectomes. Simulated connectomes are derived from random noise projected through the source localization algorithm. Regions were divided by hemisphere (L and R) and arranged anatomically from posterior (P) to anterior (A). Values on the right show, for each connectivity measure, in order: the correlation between the average real and average simulated connectome, the average of the 581 correlations between each real and simulated connectome, the correlation between the average real connectome and average edge length, and the average of the 581 correlations between each real connectome and edge length. COH: coherence; PLV: phase locking value.

**Fig. 2.**
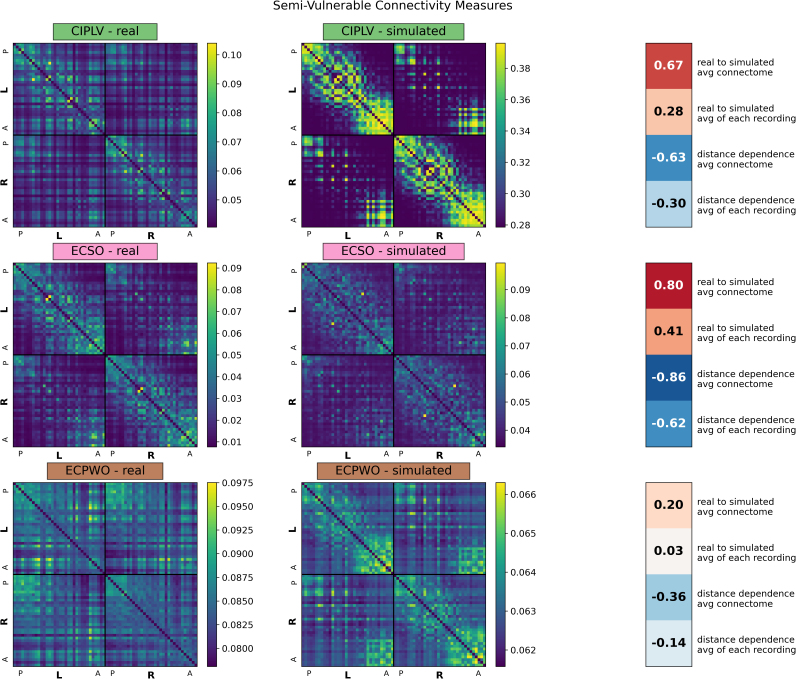
Average real and simulated broadband (2.5–45.0 Hz) connectomes for connectivity measures semi-vulnerable to volume conduction. Averages are based off of all 581 recordings across 50 participants for both real and simulated connectomes. Simulated connectomes are derived from random noise projected through the source localization algorithm. Regions were divided by hemisphere (L and R) and arranged anatomically from posterior (P) to anterior (A). Values on the right show, for each connectivity measure, in order: the correlation between the average real and average simulated connectome, the average of the 581 correlations between each real and simulated connectome, the correlation between the average real connectome and average edge length, and the average of the 581 correlations between each real connectome and edge length. CIPLV: corrected imaginary phase locking value; ECSO: envelope correlation with symmetric orthogonalization; ECPWO: envelope correlation with pairwise orthogonalization.

**Fig. 3.**
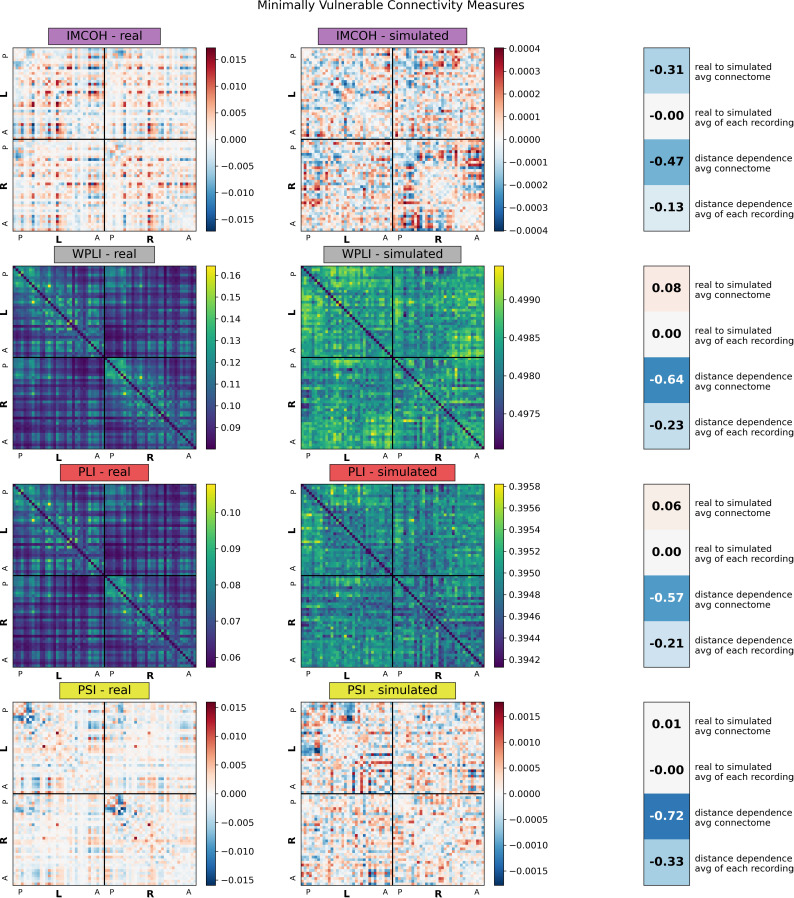
Average real and simulated broadband (2.5–45.0 Hz) connectomes for connectivity measures minimally vulnerable to volume conduction. Averages are based off of all 581 recordings across 50 participants for both real and simulated connectomes. Simulated connectomes are derived from random noise projected through the source localization algorithm. Regions were divided by hemisphere (L and R) and arranged anatomically from posterior (P) to anterior (A). Values on the right show, for each connectivity measure, in order: the correlation between the average real and average simulated connectome, the average of the 581 correlations between each real and simulated connectome, the correlation between the average real connectome and average edge length, and the average of the 581 correlations between each real connectome and edge length. IMCOH: imaginary coherence; WPLI: weighted phase lag index; PLI: phase lag index; PSI: phase slope index.

We considered the correlation between real and simulated connectomes and distance-dependence effects (Figs. 1–3). COH and PLV, the two measures without volume conduction correction, showed a strong correlation between real and simulated connectomes, averaging r = 0.78 and r = 0.76, respectively, for individual connectomes, or r = 0.90 for the averaged connectomes (Fig. 1; Vulnerable measures). Both CIPLV and ECSO showed a reduced but non-negligible similarity between real and simulated connectomes in broadband, averaging r = 0.28 and r = 0.41, respectively, suggesting some vulnerability to artifact (Fig. 2; Semi-vulnerable measures). However, these effects were much less pronounced at other frequency bands, with real-to-simulated correlations averaging r = 0.12 and r = 0.03 for alpha band. All other connectivity measures showed essentially no correlation between real and simulated connectomes (Fig. 3; Minimally vulnerable measures), though both ECPWO and IMCOH showed some real-simulated similarity when considering the averaged connectome (r = 0.20 and r = -0.31, respectively).

All connectivity measures showed some distance dependence, but the effect was strongest in COH (on average, r = -0.78), PLV (r = -0.77), and ECSO (r = -0.62), and relatively minimal in ECPWO (r = -0.14) and IMCOH (r = -0.13). All real connectomes and most simulated connectomes showed left-right hemisphere symmetry and stronger within-hemisphere than between-hemisphere connectivity (Supplemental Table S2).

### 3.2 Self-stability and participant identifiability in simulated connectomes

**Fig. 4.**
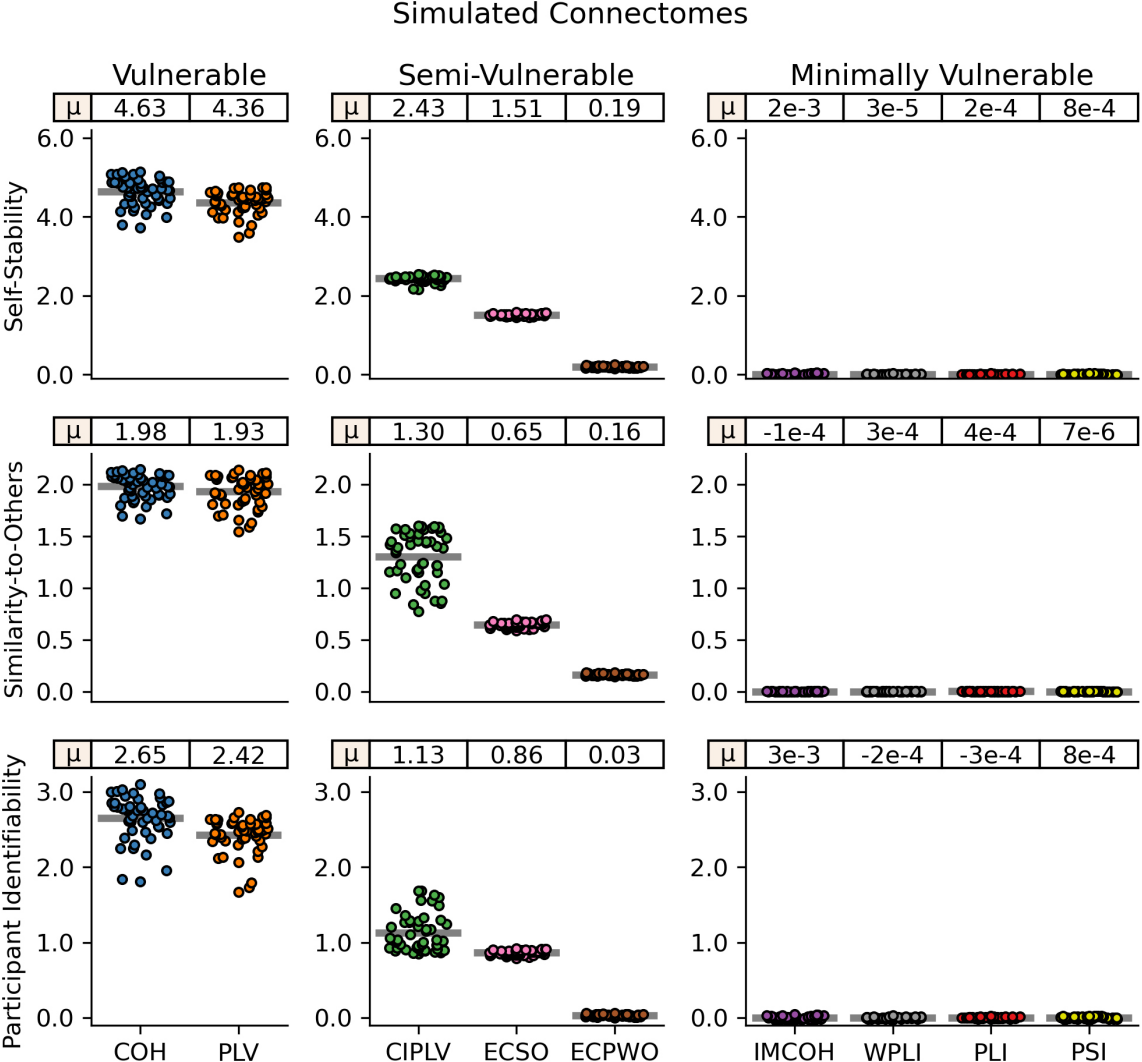
Broadband (2.5–45.0 Hz) connectome stability and identifiability across FC measures for simulated connectomes. Simulated connectomes are derived from random noise projected through the source localization algorithm. Each dot represents one participant. Lines represent mean values across participants, which are also displayed at the top of each subplot. From top to bottom, subplots represent: Mean self-stability, the average Fisher-z correlation between connectomes of the same participant. Mean similarity-to-others, the average Fisher-z correlation between a participant’s connectomes and connectomes from all other participants. Participant identifiability, the difference between mean self-stability and mean similarity-to-others. Note different y-axes across columns. COH: coherence; PLV: phase locking value; CIPLV: corrected imaginary phase locking value; ECSO: envelope correlation with symmetric orthogonalization; ECPWO: envelope correlation with pairwise orthogonalization; IMCOH: imaginary coherence; WPLI: weighted phase lag index; PLI: phase lag index; PSI: phase slope index.

In simulated connectomes, we found artifactually high reliability, as measured with self-stability, using the two FC measures without volume conduction mitigation, COH and PLV, measuring z = 4.63 and 4.36, respectively (Fig. 4). COH and PLV also showed high similarity-to-others (z = 1.98 and z = 1.93), suggesting the volume conduction leads to consistent artifactual connectome patterns across participants. While the effect was not as severe, CIPLV, ECSO, and ECPWO also showed artifactually high self-stability (z = 2.43, z = 1.53, z = 0.19) and similarity-to-others (z = 1.30, z = 0.65, z = 0.16), though the magnitude of this effect was minimal in ECPWO. However, for ECSO and ECPWO, the effects were not present in alpha band (z ≈ 0) and were reduced in beta band compared with broadband. IMCOH, WPLI, PLI, and PSI all showed no effect of self-stability or similarity-to-others (z ≈ 0) across frequency bands. COH, PLV, CIPLV, and ECSO had high degrees of individualization in the simulated connectomes, with high match rates (>99%) and a 100^th^ percentile for self-stability relative to similarity-to-others (Supplemental Fig. S1). IMCOH, WPLI, PLI, and PSI again showed no matching (<1%) and ~50^th^ percentile for self-stability scores, as would be expected with no individually unique information available. While ECPWO showed no matching, its average self-stability percentile was 67%, suggesting some artifactual influence captured in the simulated connectomes.

### 3.3 Self-stability and participant identifiability of real connectomes

**Fig. 5.**
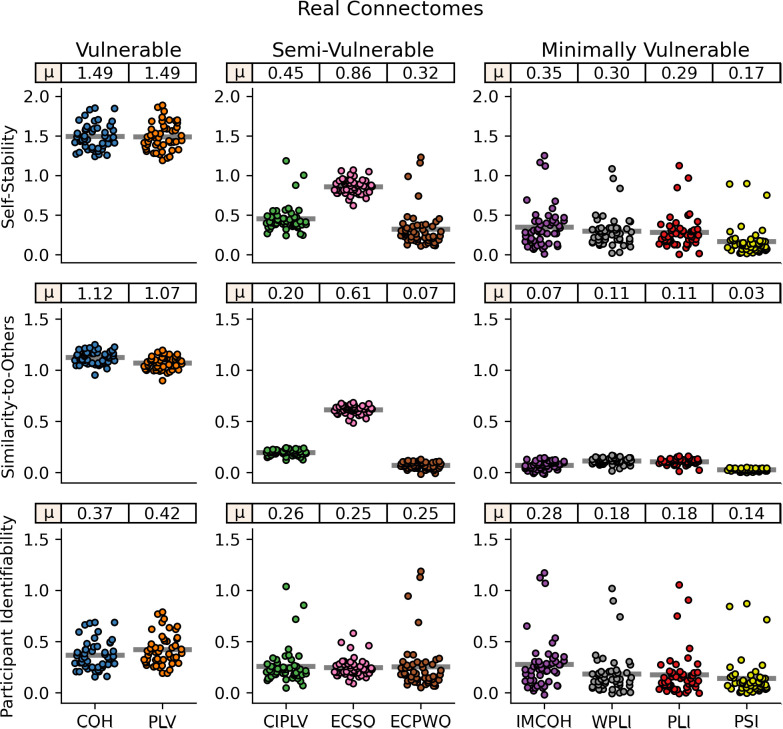
Broadband (2.5–45.0 Hz) connectome stability and identifiability across FC measures for real connectomes. Each dot represents one participant. Lines represent mean values across participants, which are also displayed at the top of each subplot. From top to bottom, subplots represent: Mean self-stability, the average Fisher-z correlation between connectomes of the same participant, collected on different days. Mean similarity-to-others, the average Fisher-z correlation between a participant’s connectomes and connectomes from all other participants. Participant identifiability, the difference between mean self-stability and mean similarity-to-others. Note different y-axes across columns. COH: coherence; PLV: phase locking value; CIPLV: corrected imaginary phase locking value; ECSO: envelope correlation with symmetric orthogonalization; ECPWO: envelope correlation with pairwise orthogonalization; IMCOH: imaginary coherence; WPLI: weighted phase lag index; PLI: phase lag index; PSI: phase slope index.

The connectivity measures with high self-stability and identifiability of simulated connectomes also had high self-stability and identifiability of real connectomes (Fig. 5). COH and PLV showed the highest self-stability (z = 1.49) and participant identifiability (z = 0.37 and z = 0.42, respectively), followed by ECSO (self-stability z = 0.86, participant identifiability z = 0.25) and CIPLV (self-stability z = 0.45, participant identifiability z = 0.26). ECSO showed the highest match rate (40%) and self-stability percentile (98%; Supplemental Fig. S2). Of measures minimally vulnerable to artifact, IMCOH showed the highest self-stability (z = 0.35), match rate (19%), and self-stability percentile (84%). It also showed the third highest identifiability (z = 0.28), behind only COH and PLV. PSI showed the least self-stability and identifiability. Results were largely similar in alpha band and beta band compared with broadband, although CIPLV, ECSO, and ECPWO showed reduced self-stability and reduced identifiability, matching the reduced self-stability and identifiability found in their alpha and beta band simulated connectomes compared with simulated broadband. IMCOH, PLI, WPLI, and PSI also showed higher stability and identifiability in broadband compared with alpha or beta band, though the effect was much less prominent.

Across reliability and identifiability metrics, nearly all differences between FC measures were significant (uncorrected p < 0.005; Supplemental Fig. S3). This occurred even if mean differences were small due to the use of paired tests with changes between FC measures relatively consistent across participants (i.e., the most individualized participants were generally the same for all FC measures).

### 3.4 Recording length and reliability

**Fig. 6.**
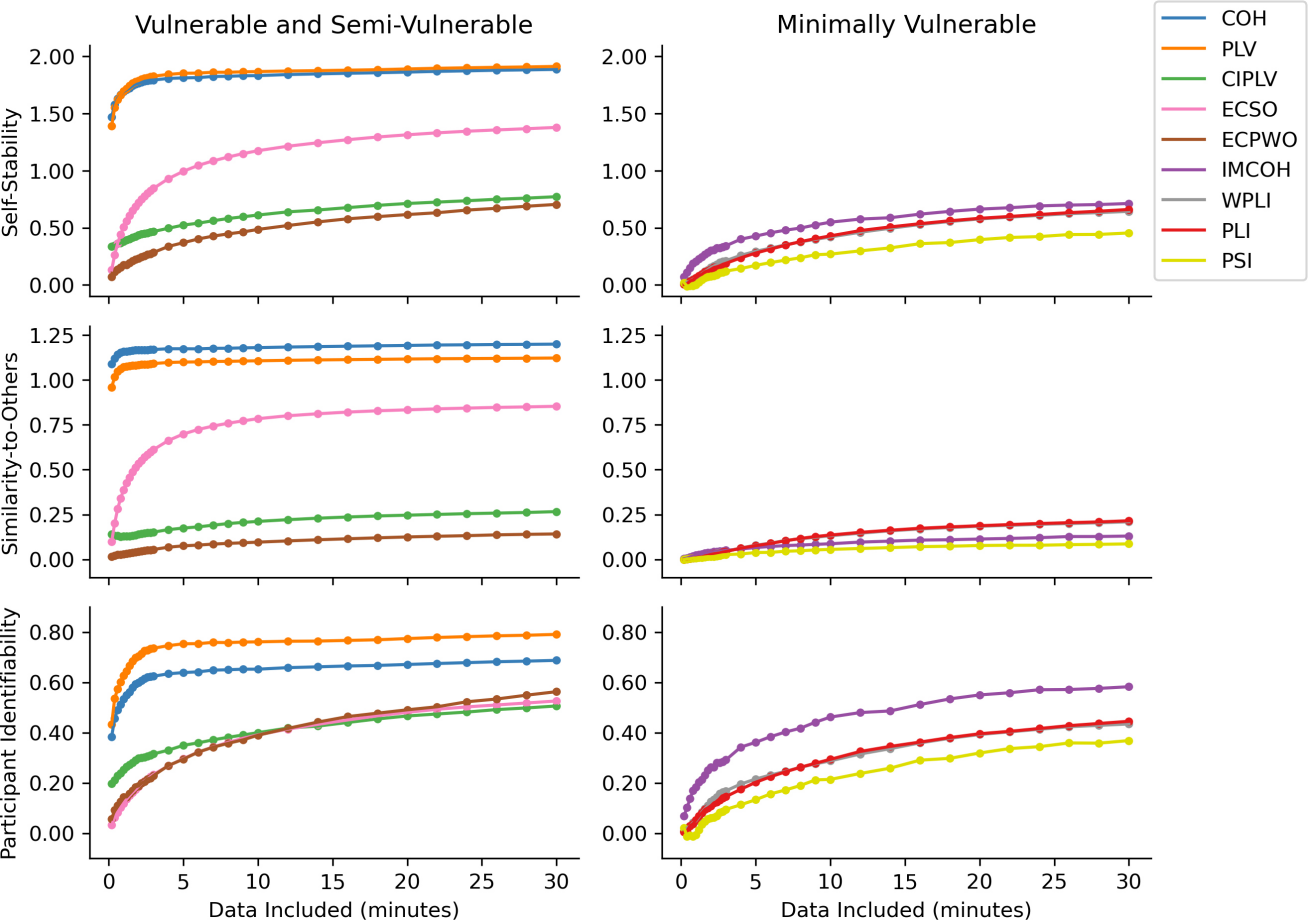
Effect of different recording lengths on self-stability and identifiability, across FC measures, for broadband (2.5–45.0 Hz) connectivity. Points shown are across-participant averages. For each participant, the recordings from their first two sessions and from their last two sessions were concatenated, giving two sets of data per participant. Data included refer to the total amount of data in each set, for example, “12 minutes included” would refer to using the first 2 minutes available from each recording, six recordings in the first two sessions and six in the last two sessions. From top to bottom, subplots represent: Mean self-stability, the average Fisher-z correlation between connectomes of the same participant, averaged across participants. Mean similarity-to-others, the average Fisher-z correlation between a participant’s connectomes and connectomes from all other participants, averaged across participants. Mean participant identifiability, the difference between self-stability and mean similarity-to-others, averaged across participants. COH: coherence; PLV: phase locking value; CIPLV: corrected imaginary phase locking value; ECSO: envelope correlation with symmetric orthogonalization; ECPWO: envelope correlation with pairwise orthogonalization; IMCOH: imaginary coherence; WPLI: weighted phase lag index; PLI: phase lag index; PSI: phase slope index.

We assessed the effect of recording length on self-stability and identifiability. COH and PLV reached peak self-stability with relatively short quantities of data (~2 minutes; Fig. 6). Though more data were required to reach an asymptote for participant identifiability, there was negligible benefit for more than ~5 minutes. However, measures that implemented at least some volume conduction mitigation continued to show benefit with longer and longer recordings, up to 30 minutes. Regardless of recording length, PSI, PLI, and WPLI tended to show the lowest reliability, suggesting longer recording lengths are insufficient to improve reliability of these measures.

### 3.5 Reliability of age-group effects as a function of duration

**Fig. 7.**
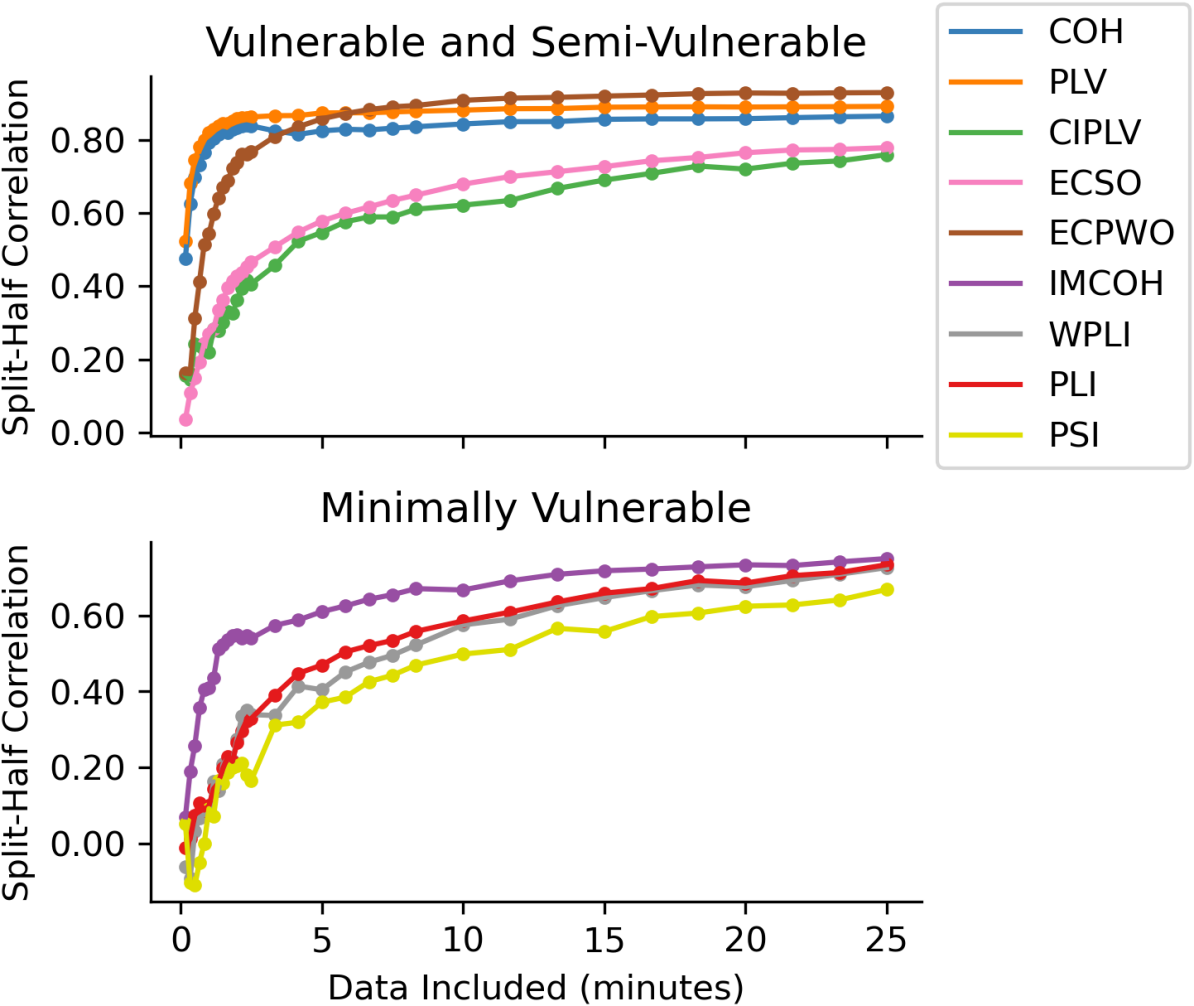
Effect of different recording lengths on the stability of age-group effects across recording sessions for broadband (2.5–45.0 Hz) connectivity. For each participant, the recordings from their first two sessions and from their last two sessions were concatenated, giving two sets of data per participant, from which connectomes were generated. Across parent–child pairs, using each participant’s first set of data, paired t-tests were used to calculate t values of age-group effects for each connectivity edge. This was repeated for the second set of data, and the two sets of t values were then correlated, to assess the stability of measured age-group effects. Data included refer to the total amount of data in each set, for example, “10 minutes included” would refer to using the first 2 minutes available from each recording, five recordings in the first two sessions and five in the last two sessions. COH: coherence; PLV: phase locking value; CIPLV: corrected imaginary phase locking value; ECSO: envelope correlation with symmetric orthogonalization; ECPWO: envelope correlation with pairwise orthogonalization; IMCOH: imaginary coherence; WPLI: weighted phase lag index; PLI: phase lag index; PSI: phase slope index.

Reliability of age-group effects between children and adults showed a similar pattern as identifiability (Fig. 7). COH and PLV reached high correlations (r > 0.8) with relatively short quantities of data, while the other measures continued to show increasing reliability of age-group effects with more data included. Of the measures less sensitive to volume conduction, IMCOH again performed the best, while PSI performed the worst. In general, there was more variance between measures at different frequency bands, with the range between the best-performing and worst-performing measures being larger at alpha and beta band than at broadband. However, all FC measures showed high age-group sensitivity consistency (r > 0.4; r > 0.6 excluding alpha-band PSI) with sufficient data.

### 3.6 Effect of forward solution for generating simulated data

The impact of volume conduction could be affected by using the same forward solution to both project simulated time courses from source space to sensor space and to project them back to source space. Supplemental Table S3 shows the comparison between using the same forward solution twice and using different forward solutions, where different conductivity values were assumed when moving from source to sensor space and different spacing for source space. Both approaches resulted in highly similar outputs, with near identical correlations to real data. For measures vulnerable or semi-vulnerable to volume conduction, the two types of connectomes were highly correlated with each other, more so than either approach to the real connectomes. For the measures minimally vulnerable to volume conduction, the two types of simulated connectomes had near zero correlation to each other or to real data. This demonstrates that using different forward solutions does not materially alter the simulated connectomes or their relationship with real data in our analyses.

## 4 Discussion

In this study, we investigated the reliability of commonly used EEG FC measures for whole-brain connectomics. We found that for a set of measures with no or minimal correction for volume conduction, simulated data with no true underlying connectivity showed relatively strong correlations with real connectomes. This anatomical dependency leads to inflated reliability metrics at the expense of validity. For this reason, we defined three groups of measures: measures vulnerable to volume conduction (COH and PLV), semi-vulnerable (ECSO, ECPWO, and CIPLV), and minimally vulnerable (IMCOH, PSI, PLI, and WPLI). We consider the anatomically sensitive measures to have relatively lower validity than the latter anatomically insensitive measures. Among the minimally vulnerable measures, IMCOH showed the highest identifiability and the highest reproducibility of age-group effects across split halves. We further find that for these measures, both identifiability and test–retest reproducibility of age-group effects continued to improve at 25–30 minutes of data, while the more vulnerable measures reach asymptotic reliability earlier. Together our results can support study design in EEG connectomics research, demonstrating trade-offs between reliability and validity and providing guidance on decisions regarding data collection duration.

Simulations in which independent signals were run through individual head geometry showed that there are varying degrees of anatomical dependence across different EEG functional connectivity measures. COH and PLV do not attempt to correct for volume conduction and show the highest correlation between individual real and simulated no-connectivity connectomes, and in turn these measures show the highest test–retest self-stability and individualization, and shortest duration to reach asymptotic reliability of connectomes and age-group effects. Colclough et al. (2016) found similar reliability findings in MEG, where FC measures prone to volume conduction had higher within-subject and between-subject consistency, which they suggest is primarily due to volume conduction artifacts. For this reason, they argue against using these measures, suggesting that “interpretable connectivity estimation is only possible when zero-lag connections are removed or otherwise ignored.” Our results add to these concerns, suggesting that where differences between groups or cross-participant correlation effects are found, these may be largely driven by head geometry rather than connectivity per se, and should, therefore, be interpreted with caution.

ECSO, CIPLV, and ECPWO show more moderate levels of anatomical sensitivity, indicated by self-stability of simulated no-connectivity connectomes. Self-stability and identifiability of these metrics are lower than COH and PLV, indicating partial but incomplete correction for volume conduction. If we consider high individualization of real connectomes alongside low identifiability of simulated connectomes as desirable properties, ECPWO has the advantage among these three, especially in broadband, though still with less identifiability than IMCOH.

Finally, IMCOH, PSI, PLI, and WPLI show negligible vulnerability to volume conduction, which increases confidence in using identifiability as a measure of reliability. With these measures, there is also likely a validity trade-off in that there is a risk of over-correcting and attenuating “true” connectivity. Indeed, the developers of PLI (Stam et al., 2007) note that their approach requires accepting the risk of missing meaningful interactions that have near zero phase coherence and acknowledging that the remaining connectivity information may be incomplete. Similarly, the developers of IMCOH (Nolte et al., 2004) refer to IMCOH as an “extreme position” that contains, “at best, only half of the picture”. An intracranial EEG study, where volume conduction is less prominent, suggested that measures designed to ignore zero-phase lag coherence showed less alignment with structural connectivity than PLV (Messé et al., 2023). These previous results suggest these measures may fail to capture sufficient information with typical short duration recordings, and thus when volume conduction is less of a concern, PLV or COH may be preferable. We further note that although these measures were originally proposed to be entirely artifact free, some work suggests they can still be susceptible to false positives due to field spread (Palva et al., 2018).

Of the FC measures with negligible anatomical influence, IMCOH outperformed PSI, PLI, and WPLI on metrics assessed here. IMCOH showed higher self-stability, identifiability, and reliability of age-group effects across durations. Of phase-based methods that correct for volume conduction, the use of IMCOH is emerging as the *de facto* standard for full connectome analyses (Nentwich et al., 2020; Wirsich et al., 2021). Given our results, and the need for reproducibility across studies, this convergence seems appropriate.

PSI is the one effective connectivity measure included here. While relative to other measures the reliability of connectomes and age-group effects was generally lower, there may nonetheless be important conceptual reasons for choosing this measure. Our findings suggest that reliability of this (and other) measures can be improved with longer scan durations.

Of the two envelope correlation measures tested, we found that ECPWO outperformed ECSO, both with less sensitivity to artifact and in terms of identifiability. It is worth noting that while both ECPWO and ECSO demonstrated sensitivity to artifact in broadband, the effect was diminished in narrower bands, being nearly negligible in alpha band. This suggests their utility may be greater when considering narrower frequency ranges. While ECPWO outperformed ECSO based on metrics used here, there may be theoretical reasons to prefer ECSO, as correcting all edges to the same degree may allow for better estimates of network effects (Colclough et al., 2015). Given that envelope correlations are thought to capture different information than phase-based connectivity measures (Nentwich et al., 2020), there may be other reasons to prefer these measures rather than IMCOH or other measures used here.

Considering performance in relation to data quantity, we found that COH and PLV required far shorter recordings than other measures to reach an identifiability asymptote (i.e., ~90 epochs, 3 minutes). However, for standard recording lengths, for example, 5 minutes (here 150 epochs), IMCOH and other measures that correct for volume conduction may have less than half of the maximum identifiability a longer recording would provide. Our results suggest that for whole-brain connectomics research, relatively long quantities of data may prove beneficial to reaching stable values for anatomically insensitive FC measures.

Strengths of this study include a unique dataset with long time series and two groups, and a combination of simulations and reliability analyses for context. We also note several limitations, in particular areas where design decisions may limit the generalizability of findings. We estimated FC in source space, which is less affected by volume conduction compared with sensor space (Nolte et al., 2004; Sadaghiani et al., 2022), and findings may not generalize to sensor space or other parcellations. We split recordings into 2-second epochs for all analyses, though longer epochs may more strongly favor connectivity measures that discard more of the signal, such as IMCOH. While there is some evidence that longer epochs are preferential in general for calculating FC (Nagy et al., 2024), this requires one to consider the trade-off of excluding more data or less stringent artifact correction, which may require special consideration in data collected from children. Additionally, we used a 64-channel sensor net; a 128- or 256-channel device may show different sensitivity. Data were collected during passive viewing conditions, which may differ from task-free “rest”. Many other methodological decisions made here may have impacted our findings, such as source localization algorithm, artifact removal approach, and use of multitaper spectrum estimation. Many additional EEG FC measures also exist (Cao et al., 2022), and other measures may have better trade-offs between retaining signal and removing noise. Future work should compare more connectivity measures and more directly compare different ways to correct for volume conduction and other artifacts. Study findings may be specific to the dataset and processing approaches used here. Studies in different datasets are needed to confirm generalizability of findings.

## 5 Conclusions

The present study investigated reliability of several commonly used EEG FC measures. We found that different measures show varying dependence on individual anatomy, inflating reliability at the expense of validity. IMCOH had the overall best performance among minimally vulnerable measures, considering individualization and reliability of group effects. We also found that all measures that include some volume correction benefited from longer acquisition times, with reliability improvements up to 25–30 minutes of data collected. Based on these findings, we recommend careful consideration of which FC measure to use and planning of data collection durations to achieve reliability goals.

## Supplementary Material

Supplementary Material

## Data Availability

Python scripts and specific details of the videos watched are available at: https://github.com/BrayNeuroimagingLab/BNL_open/tree/main/EEG_Preprocessing_and_Analysis. Connectomes and raw EEG or MRI data are available upon request.
