# Supplementary material for: Towards precision EEG connectomics: Evaluating the benefits of dense sampling
Source: Imaging Neurosci (Camb). 2026 Jun 4;4:IMAG.a.1245. doi: 10.1162/IMAG.a.1245 (PMC13237998; doi:10.1162/IMAG.a.1245)
Supplement: Supplementary Material [file IMAG.a.1245_supp.pdf]

## Supplemental Figures and Tables

**Supplemental Table 1. Age, sex, and visit timing of participants.**

| Family | Parent | | Child | | Days after visit 1 | | |
|---|---|---|---|---|---|---|---|
| | Sex | Age (Visit 1; years) | Sex | Age (Visit 1; years) | Visit 2 | Visit 3 | Visit 4 |
| 1 | F | 40.44 | F | 7.46 | 11 | 14 | 22 |
| 2 | F | 36.77 | M | 7.26 | 51 | 86 | 125 |
| 3 | F | 36.78 | M | 7.21 | 53 | 111 | 467 |
| 4A | F | 39.56 | M | 8.91 | 7 | 28 | 35 |
| 4B | M | 42.05 | M | 7.34 | 7 | 28 | 147 |
| 5A | M | 44.57 | M | 8.84 | 28 | 35 | 42 |
| 5B | F | 44.04 | F | 7.22 | 7 | 49 | 63 |
| 6 | F | 45.03 | M | 7.26 | 70 | 77 | 84 |
| 7 | M | 38.03 | M | 7.62 | 78 | 118 | 126 |
| 8 | F | 44.25 | M | 8.90 | 17 | 103 | 129 |
| 9 | F | 42.00 | M | 8.70 | 77 | 92 | 108 |
| 10 | F | 44.92 | M | 8.07 | 81 | 116 | 145 |
| 11 | M | 36.77 | M | 7.57 | 7 | 14 | 42 |
| 12 | F | 40.22 | F | 7.94 | 56 | 109 | 182 |
| 13 | M | 43.60 | M | 8.02 | 7 | 21 | 34 |
| 14 | M | 46.94 | F | 7.60 | 28 | 35 | 98 |
| 15 | M | 42.63 | F | 8.55 | 7 | 14 | 35 |
| 16 | M | 33.75 | F | 8.26 | 23 | 26 | 50 |
| 17 | F | 47.13 | F | 8.82 | 7 | 36 | 41 |
| 18 | M | 41.73 | F | 8.48 | 28 | 35 | 42 |
| 19 | M | 42.12 | F | 7.98 | 7 | 13 | 20 |
| 20 | F | 44.46 | F | 8.49 | 7 | 45 | 59 |
| 21 | M | 38.09 | F | 7.03 | 6 | 42 | 49 |
| 22 | M | 34.88 | F | 6.90 | 7 | 14 | 21 |
| 23 | M | 43.58 | F | 6.56 | 21 | 43 | 50 |

**Supplemental Table 2. Left-right hemisphere symmetry (correlation between left-to-left and right-to-right connectivity), average within hemisphere connectivity, and average between hemisphere connectivity.** Colors represent as follows: Green > 0.9, blue > 0.7, yellow > 0.3, red < 0, grey < 5% difference.

| Frequency Band | Connectivity Measure | Data Type | Left-Right Symmetry | Within Hemisphere | Between Hemisphere | Percent Difference (Within-Between) |
|---|---|---|---|---|---|---|
| 2.5-45.0 Hz | coh | simulated | 0.995 | 0.4294 | 0.2271 | 47.04 |
| | | real | 0.993 | 0.3494 | 0.1906 | 45.51 |
| | plv | simulated | 0.995 | 0.9957 | 0.5862 | 41.18 |
| | | real | 0.992 | 0.7049 | 0.4049 | 42.56 |
| | ciplv | simulated | 0.992 | 0.319 | 0.2914 | 8.62 |
| | | real | 0.93 | 0.05612 | 0.05057 | 9.89 |
| | ecso | simulated | 0.946 | 0.04743 | 0.04199 | 11.49 |
| | | real | 0.986 | 0.02916 | 0.01961 | 32.75 |
| | ecpwo | simulated | 0.971 | 0.06332 | 0.06289 | 0.69 |
| | | real | 0.787 | 0.08407 | 0.08401 | 0.07 |
| | imcoh | simulated | 0.304 | 1.07E-04 | 1.04E-04 | 2.42 |
| | | real | 0.794 | 0.002974 | 0.002661 | 10.52 |
| | wpli | simulated | -3.93E-03 | 0.4984 | 0.4984 | 0.00 |
| | | real | 0.904 | 0.1066 | 0.09817 | 7.94 |
| | pli | simulated | 0.103 | 0.3949 | 0.3949 | -0.01 |
| | | real | 0.881 | 0.07202 | 0.0674 | 6.41 |
| | psi | simulated | -0.235 | 4.13E-04 | 3.92E-04 | 5.09 |
| | | real | 0.732 | 0.002315 | 0.001733 | 25.18 |
| 8.0-13.0 Hz | coh | simulated | 0.995 | 0.4322 | 0.2282 | 47.20 |
| | | real | 0.993 | 0.3914 | 0.2321 | 40.62 |
| | plv | simulated | 0.995 | 1.026 | 0.6012 | 41.42 |
| | | real | 0.992 | 0.783 | 0.489 | 37.55 |
| | ciplv | simulated | 0.992 | 0.3244 | 0.2957 | 8.88 |
| | | real | 0.868 | 0.05839 | 0.05395 | 7.60 |
| | ecso | simulated | 0.811 | 0.07871 | 0.07836 | 0.45 |
| | | real | 0.986 | 0.02396 | 0.01888 | 21.20 |
| | ecpwo | simulated | 0.348 | 0.1526 | 0.1526 | 0.00 |
| | | real | 0.804 | 0.165 | 0.1649 | 0.06 |
| | imcoh | simulated | -0.281 | 2.88E-04 | 2.68E-04 | 7.12 |
| | | real | 0.75 | 0.004437 | 0.004174 | 5.93 |
| | wpli | simulated | 0.13 | 0.5216 | 0.5217 | -0.02 |
| | | real | 0.807 | 0.1107 | 0.1045 | 5.57 |
| | pli | simulated | 0.121 | 0.4029 | 0.403 | -0.02 |
| | | real | 0.781 | 0.07445 | 0.07123 | 4.33 |
| | psi | simulated | 0.198 | 1.78E-04 | 1.25E-04 | 30.13 |
| | | real | 0.462 | 6.87E-04 | 5.66E-04 | 17.62 |
| 13.0-30.0 Hz | coh | simulated | 0.995 | 0.4301 | 0.2274 | 47.20 |
| | | real | 0.993 | 0.3507 | 0.1896 | 45.91 |
| | plv | simulated | 0.995 | 1.003 | 0.5895 | 41.18 |
| | | real | 0.992 | 0.7142 | 0.4098 | 42.57 |
| | ciplv | simulated | 0.992 | 0.3202 | 0.2923 | 8.71 |
| | | real | 0.885 | 0.05793 | 0.05251 | 9.36 |
| | ecso | simulated | 0.931 | 0.04603 | 0.0449 | 2.45 |
| | | real | 0.989 | 0.02544 | 0.01662 | 34.67 |
| | ecpwo | simulated | 0.924 | 0.0915 | 0.09133 | 0.19 |
| | | real | 0.785 | 0.1039 | 0.1035 | 0.33 |
| | imcoh | simulated | 0.144 | 1.41E-04 | 1.63E-04 | -15.57 |
| | | real | 0.684 | 0.003785 | 0.003516 | 7.13 |
| | wpli | simulated | -0.0902 | 0.5029 | 0.5028 | 0.01 |
| | | real | 0.819 | 0.1077 | 0.09942 | 7.73 |
| | pli | simulated | -1.35E-03 | 0.3965 | 0.3965 | 0.01 |
| | | real | 0.789 | 0.07382 | 0.06942 | 5.96 |
| | psi | simulated | 0.105 | 2.90E-04 | 2.67E-04 | 7.94 |
| | | real | 0.856 | 0.001616 | 0.001014 | 37.25 |

**Supplemental Table 3. Comparison of using the same forward solution and different forward solutions for generating simulated data.** A forward solution was generated for projecting real data from sensor space to source space. Simulated data was generated from random noise for each node and projected to source space, using either the same forward solution as for real data (**same fwd**) or one with alternative connectivity assumptions and different spacing for source space (**diff fwd**), before being projected back to source space using the original forward solution. For each frequency band and connectivity measure, an averaged connectome was generated for each individual (**individual**) or for the entire sample across all participants (**avg**). Vectorized connectomes were compared with Pearson correlation. For connectomes from individuals, averages and standard deviations of correlations across participants are shown. Color coding reflects vulnerability to volume conduction.

| Band | Measure | Real to sim same fwd | | Real to sim diff fwd | | Sim same fwd to diff fwd | |
|---|---|---|---|---|---|---|---|
| | | Individual | Avg | Individual | Avg | Individual | Avg |
| 2.5-45.0 Hz | coh | 0.781 (0.05) | 0.838 | 0.798 (0.046) | 0.86 | 0.989 (0.006) | 0.994 |
| | plv | 0.768 (0.053) | 0.84 | 0.779 (0.051) | 0.856 | 0.988 (0.008) | 0.995 |
| | ciplv | 0.353 (0.144) | 0.422 | 0.357 (0.138) | 0.432 | 0.957 (0.036) | 0.989 |
| | ecso | 0.522 (0.05) | 0.679 | 0.532 (0.056) | 0.687 | 0.956 (0.007) | 0.98 |
| | ecpwo | -0.03 (0.147) | -0.079 | 0.022 (0.122) | -0.001 | 0.714 (0.017) | 0.924 |
| | imcoh | 0.059 (0.186) | -0.034 | 0.012 (0.138) | 0.181 | 0.044 (0.115) | 0.168 |
| | wpli | -0.033 (0.087) | -0.083 | -0.002 (0.054) | 0.125 | -0.02 (0.025) | 0.133 |
| | pli | -0.032 (0.055) | -0.099 | -0.007 (0.049) | 0.087 | -0.033 (0.028) | 0.055 |
| | psi | -0.021 (0.102) | 0.008 | -0.038 (0.067) | 0.021 | 0.014 (0.068) | -0.111 |
| 8.0-13.0 Hz | coh | 0.75 (0.057) | 0.816 | 0.768 (0.058) | 0.838 | 0.989 (0.006) | 0.994 |
| | plv | 0.73 (0.07) | 0.813 | 0.741 (0.072) | 0.829 | 0.988 (0.008) | 0.995 |
| | ciplv | 0.277 (0.095) | 0.29 | 0.272 (0.1) | 0.292 | 0.951 (0.034) | 0.989 |
| | ecso | 0.173 (0.029) | 0.419 | 0.17 (0.033) | 0.418 | 0.155 (0.024) | 0.478 |
| | ecpwo | -0.001 (0.061) | -0.021 | -0.033 (0.043) | -0.092 | 0.01 (0.036) | 0.063 |
| | imcoh | 0.068 (0.101) | 0.104 | -0.066 (0.127) | -0.046 | -0.022 (0.11) | -0.08 |
| | wpli | -0.015 (0.055) | -0.099 | -0.067 (0.066) | -0.031 | -0.013 (0.079) | -0.014 |
| | pli | 0.0 (0.044) | -0.091 | -0.047 (0.066) | 0.033 | 0.007 (0.08) | -0.019 |
| | psi | 0.001 (0.065) | 0.187 | 0.001 (0.086) | 0.049 | -0.102 (0.092) | -0.124 |
| 13.0-30.0 Hz | coh | 0.781 (0.054) | 0.84 | 0.799 (0.05) | 0.862 | 0.989 (0.006) | 0.994 |
| | plv | 0.766 (0.058) | 0.84 | 0.777 (0.057) | 0.856 | 0.988 (0.008) | 0.995 |
| | ciplv | 0.321 (0.138) | 0.346 | 0.328 (0.132) | 0.356 | 0.955 (0.036) | 0.989 |
| | ecso | 0.448 (0.052) | 0.632 | 0.444 (0.055) | 0.633 | 0.815 (0.013) | 0.947 |
| | ecpwo | -0.005 (0.077) | -0.05 | 0.03 (0.062) | 0.006 | 0.31 (0.048) | 0.729 |
| | imcoh | 0.029 (0.164) | 0.114 | 0.015 (0.105) | 0.071 | -0.02 (0.117) | 0.02 |
| | wpli | -0.045 (0.095) | -0.13 | 0.028 (0.078) | 0.145 | 0.019 (0.04) | 0.068 |
| | pli | -0.052 (0.08) | -0.127 | 0.018 (0.065) | 0.067 | 0.013 (0.037) | 0.139 |
| | psi | -0.02 (0.054) | -0.093 | -0.03 (0.051) | -0.082 | -0.014 (0.062) | 0.007 |

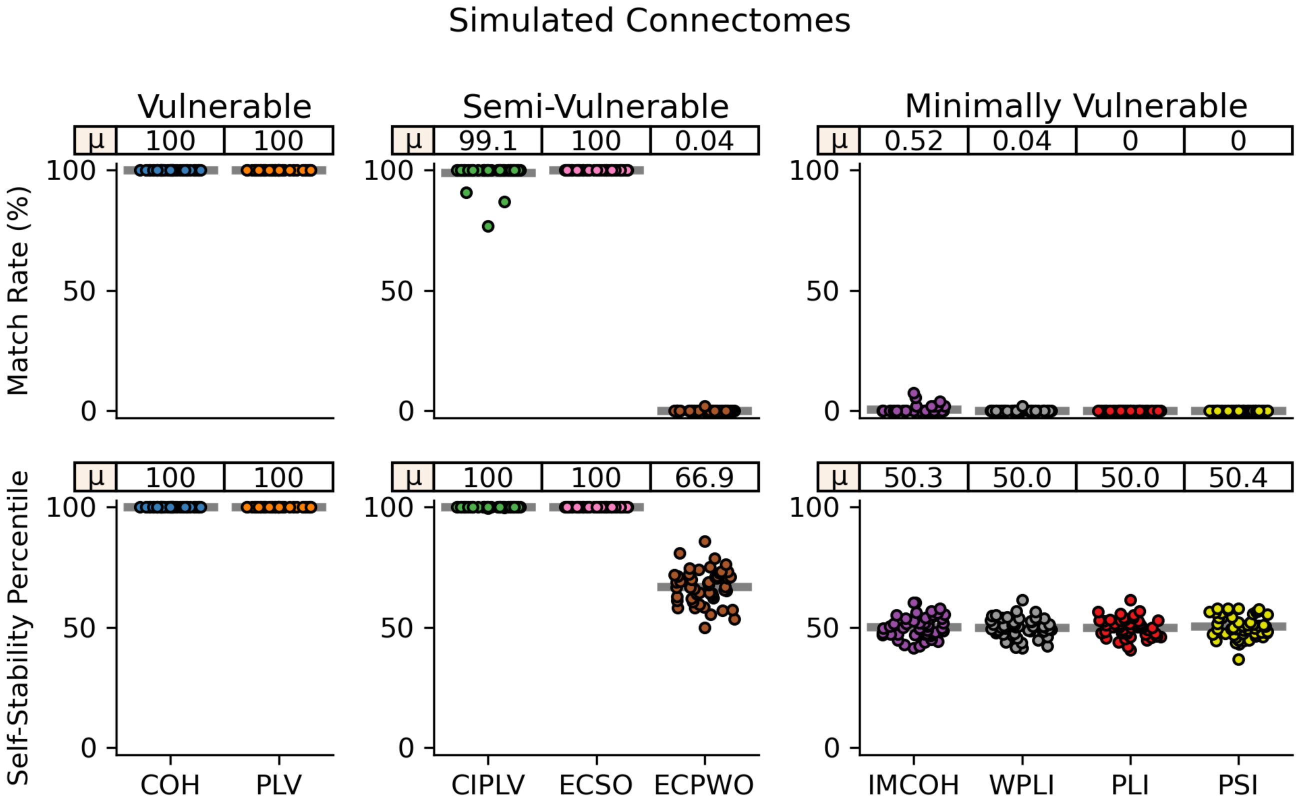


**Supplemental Figure 1. Additional broadband (2.5-45.0 Hz) identifiability metrics across FC measures for simulated connectomes.** Simulated connectomes are derived from random noise projected through the source localization algorithm. Each dot represents one participant. Lines represent mean values across participants, which are also displayed at the top of each subplot. From top to bottom, subplots represent: **Match rate**, the percentage of self-stability scores that were higher than all similarity-to-others scores. **Self-stability percentile**, the average percentile of self-stability scores, relative to similarity-to-others. *COH: coherence; PLV: phase locking value; CIPLV: corrected imaginary phase locking value; ECSO: envelope correlation with symmetric orthogonalization; ECPWO: envelope correlation with pairwise orthogonalization; IMCOH: imaginary coherence; WPLI: weighted phase lag index; PLI: phase lag index; PSI: phase slope index.*

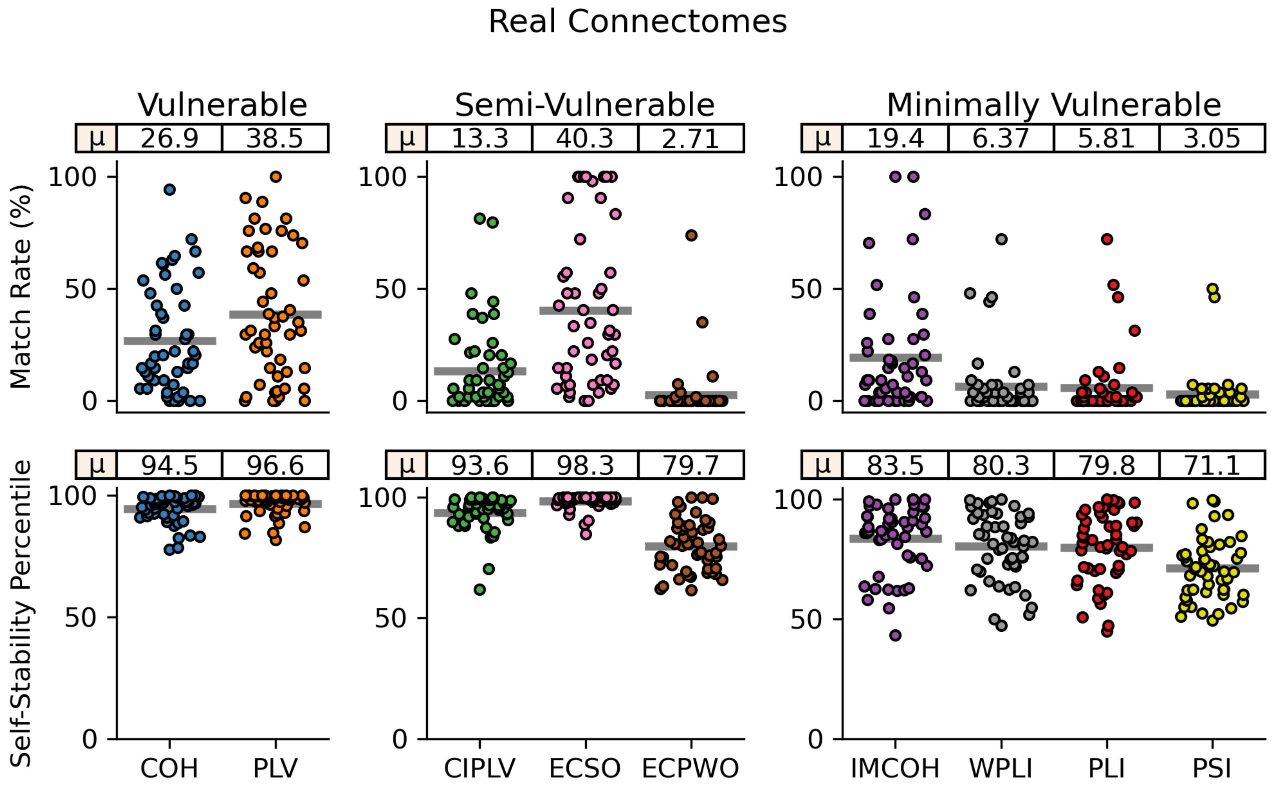


**Supplemental Figure 2. Additional broadband (2.5-45.0 Hz) identifiability metrics across FC measures for real connectomes.** Each dot represents one participant. Lines represent mean values across participants, which are also displayed at the top of each subplot. From top to bottom, subplots represent: **Match rate**, the percentage of self-stability scores that were higher than all similarity-to-others scores. **Self-stability percentile**, the average percentile of self-stability scores, relative to similarity-to-others. *COH: coherence; PLV: phase locking value; CIPLV: corrected imaginary phase locking value; ECSO: envelope correlation with symmetric orthogonalization; ECPWO: envelope correlation with pairwise orthogonalization; IMCOH: imaginary coherence; WPLI: weighted phase lag index; PLI: phase lag index; PSI: phase slope index.*

**Self-Stability**

| | PLV | CIPLV | ECSO | ECPWO | IMCOH | WPLI | PLI | PSI |
|---|---|---|---|---|---|---|---|---|
| COH | p: 0.24 | p: 7.0e-39 | p: 3.3e-37 | p: 1.8e-36 | p: 7.0e-33 | p: 4.4e-38 | p: 1.8e-37 | p: 3.2e-41 |
| PLV | | p: 1.8e-38 | p: 1.0e-35 | p: 3.3e-36 | p: 1.7e-32 | p: 1.5e-37 | p: 5.1e-37 | p: 1.0e-40 |
| CIPLV | | | p: 3.1e-25 | p: 1.3e-8 | p: 1.3e-6 | p: 2.0e-24 | p: 6.0e-24 | p: 4.6e-29 |
| ECSO | | | | p: 1.9e-23 | p: 3.4e-20 | p: 1.1e-26 | p: 2.6e-26 | p: 4.5e-32 |
| ECPWO | | | | | p: 0.32 | p: 0.16 | p: 0.048 | p: 4.5e-14 |
| IMCOH | | | | | | p: 1.4e-3 | p: 8.1e-5 | p: 5.8e-12 |
| WPLI | | | | | | | p: 3.5e-3 | p: 4.4e-15 |
| PLI | | | | | | | | p: 5.0e-14 |

**Similarity-to-Others**

| | PLV | CIPLV | ECSO | ECPWO | IMCOH | WPLI | PLI | PSI |
|---|---|---|---|---|---|---|---|---|
| COH | p: 5.7e-34 | p: 3.6e-62 | p: 1.1e-45 | p: 5.1e-62 | p: 4.6e-60 | p: 6.1e-61 | p: 2.0e-60 | p: 3.6e-63 |
| PLV | | p: 1.3e-60 | p: 5.0e-42 | p: 3.4e-60 | p: 1.8e-58 | p: 4.4e-59 | p: 1.1e-58 | p: 1.1e-61 |
| CIPLV | | | p: 3.1e-48 | p: 2.2e-30 | p: 4.5e-28 | p: 2.4e-35 | p: 4.1e-37 | p: 1.9e-39 |
| ECSO | | | | p: 2.2e-51 | p: 1.3e-48 | p: 3.8e-51 | p: 2.5e-51 | p: 1.8e-56 |
| ECPWO | | | | | p: 0.97 | p: 2.5e-12 | p: 9.2e-11 | p: 2.1e-12 |
| IMCOH | | | | | | p: 8.6e-11 | p: 1.2e-9 | p: 1.6e-9 |
| WPLI | | | | | | | p: 4.9e-7 | p: 1.8e-25 |
| PLI | | | | | | | | p: 1.2e-25 |

**Participant Identifiability**

| | PLV | CIPLV | ECSO | ECPWO | IMCOH | WPLI | PLI | PSI |
|---|---|---|---|---|---|---|---|---|
| COH | p: 4.6e-20 | p: 2.2e-5 | p: 1.4e-12 | p: 3.4e-4 | p: 0.011 | p: 3.6e-8 | p: 3.7e-8 | p: 2.2e-11 |
| PLV | | p: 1.8e-8 | p: 3.2e-16 | p: 1.7e-6 | p: 2.0e-4 | p: 1.2e-10 | p: 1.4e-10 | p: 1.0e-13 |
| CIPLV | | | p: 0.50 | p: 0.83 | p: 0.26 | p: 1.6e-13 | p: 4.3e-13 | p: 8.4e-15 |
| ECSO | | | | p: 0.76 | p: 0.29 | p: 5.6e-3 | p: 3.9e-3 | p: 1.8e-6 |
| ECPWO | | | | | p: 0.27 | p: 3.3e-4 | p: 7.7e-5 | p: 2.3e-9 |
| IMCOH | | | | | | p: 1.9e-9 | p: 9.9e-11 | p: 3.5e-10 |
| WPLI | | | | | | | p: 0.064 | p: 9.3e-5 |
| PLI | | | | | | | | p: 5.8e-4 |

**Match Rate (%)**

| | PLV | CIPLV | ECSO | ECPWO | IMCOH | WPLI | PLI | PSI |
|---|---|---|---|---|---|---|---|---|
| COH | p: 1.1e-9 | p: 5.0e-4 | p: 6.4e-4 | p: 1.8e-8 | p: 0.12 | p: 4.4e-7 | p: 1.8e-7 | p: 6.9e-9 |
| PLV | | p: 2.2e-7 | p: 0.64 | p: 5.5e-11 | p: 5.2e-4 | p: 6.6e-10 | p: 3.4e-10 | p: 2.5e-11 |
| CIPLV | | | p: 3.9e-6 | p: 4.6e-5 | p: 0.037 | p: 3.3e-5 | p: 4.9e-6 | p: 1.0e-5 |
| ECSO | | | | p: 7.2e-11 | p: 1.1e-4 | p: 1.9e-9 | p: 1.8e-9 | p: 1.9e-10 |
| ECPWO | | | | | p: 1.2e-6 | p: 0.024 | p: 0.044 | p: 0.64 |
| IMCOH | | | | | | p: 3.5e-6 | p: 3.1e-6 | p: 1.2e-6 |
| WPLI | | | | | | | p: 0.21 | p: 0.011 |
| PLI | | | | | | | | p: 0.019 |

**Self-Stability Percentile**

| | PLV | CIPLV | ECSO | ECPWO | IMCOH | WPLI | PLI | PSI |
|---|---|---|---|---|---|---|---|---|
| COH | p: 3.7e-11 | p: 0.23 | p: 7.7e-7 | p: 6.8e-14 | p: 9.6e-7 | p: 1.2e-9 | p: 1.7e-9 | p: 5.5e-17 |
| PLV | | p: 1.7e-4 | p: 4.3e-3 | p: 6.8e-16 | p: 1.0e-8 | p: 2.4e-11 | p: 3.2e-11 | p: 7.3e-19 |
| CIPLV | | | p: 2.0e-6 | p: 1.8e-11 | p: 4.0e-6 | p: 1.7e-10 | p: 3.9e-10 | p: 2.5e-16 |
| ECSO | | | | p: 9.5e-17 | p: 7.2e-10 | p: 2.1e-12 | p: 3.3e-12 | p: 3.1e-19 |
| ECPWO | | | | | p: 0.083 | p: 0.77 | p: 0.96 | p: 5.0e-6 |
| IMCOH | | | | | | p: 0.026 | p: 8.4e-3 | p: 4.1e-9 |
| WPLI | | | | | | | p: 0.35 | p: 1.5e-7 |
| PLI | | | | | | | | p: 7.1e-7 |

**Supplemental Figure 3. Statistical comparisons for broadband (2.5-45.0 Hz) connectivity between self-stability and identifiability measures, as presented in Figure 5 and Supplemental Figure 2.** Between each FC measure, a paired t-test was used to assess the statistical difference between participants' scores. Yellow shading = $p < 0.05$ Bonferroni corrected; green shading = $p < 0.05$ uncorrected. *COH: coherence; PLV: phase locking value; CIPLV: corrected imaginary phase locking value; ECSO: envelope correlation with symmetric orthogonalization; ECPWO: envelope correlation with pairwise orthogonalization; IMCOH: imaginary coherence; WPLI: weighted phase lag index; PLI: phase lag index; PSI: phase slope index.*

## Alpha Band Results

In the main body of the paper, results were presented for broadband connectivity. Here, results are presented for **alpha band connectivity (8.0 - 13.0 Hz)**. For ease of comparison, figures are labelled as either 'Figure' or 'Supplemental Figure' to match their designation elsewhere.

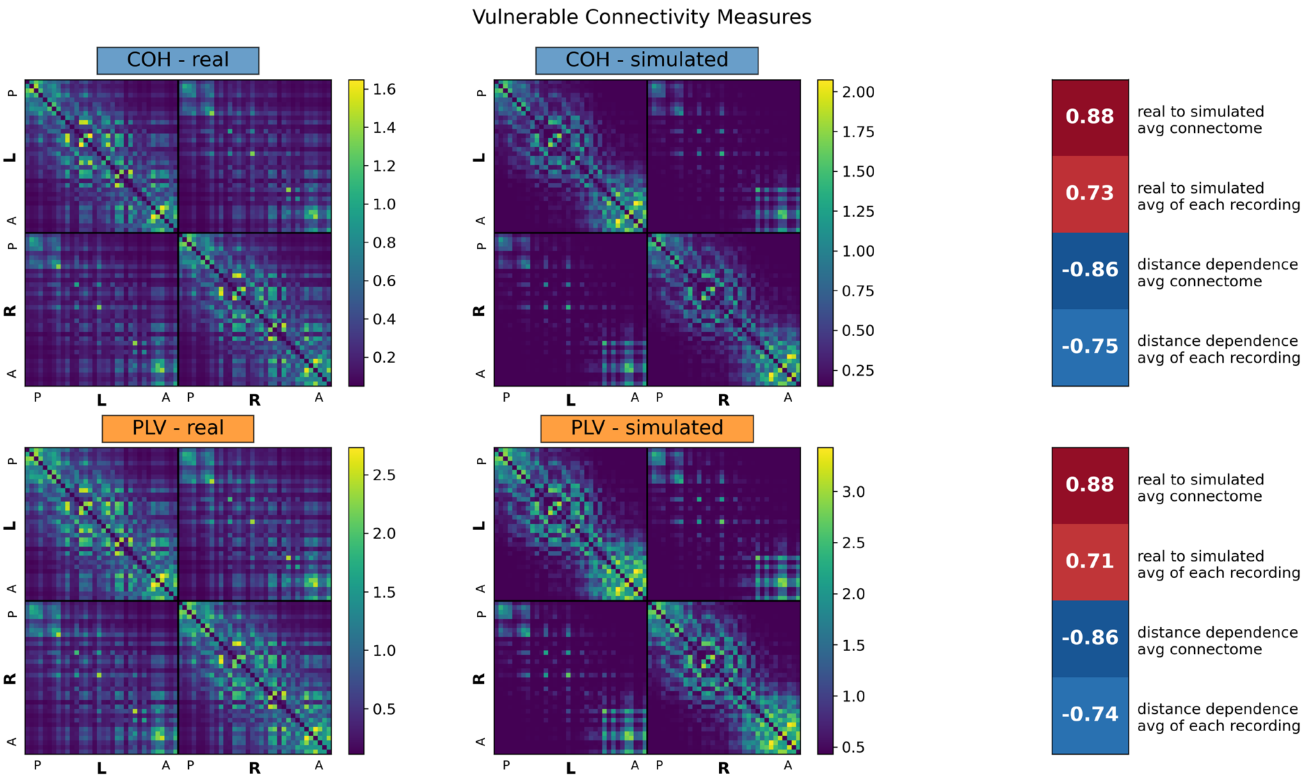


**Figure 1. Average real and simulated alpha band (8-13 Hz) connectomes for connectivity measures vulnerable to volume conduction.** Averages are based off of all 581 recordings across 50 participants for both real and simulated connectomes. Simulated connectomes are derived from random noise projected through the source localization algorithm. Regions were divided by hemisphere (L and R) and arranged anatomically from posterior (P) to anterior (A). Values on the right show, for each connectivity measure, in order: the correlation between the average real and average simulated connectome, the average of the 581 correlations between each real and simulated connectome, the correlation between the average real connectome and average edge length, and the average of the 581 correlations between each real connectome and edge length. *COH: coherence; PLV: phase locking value.*

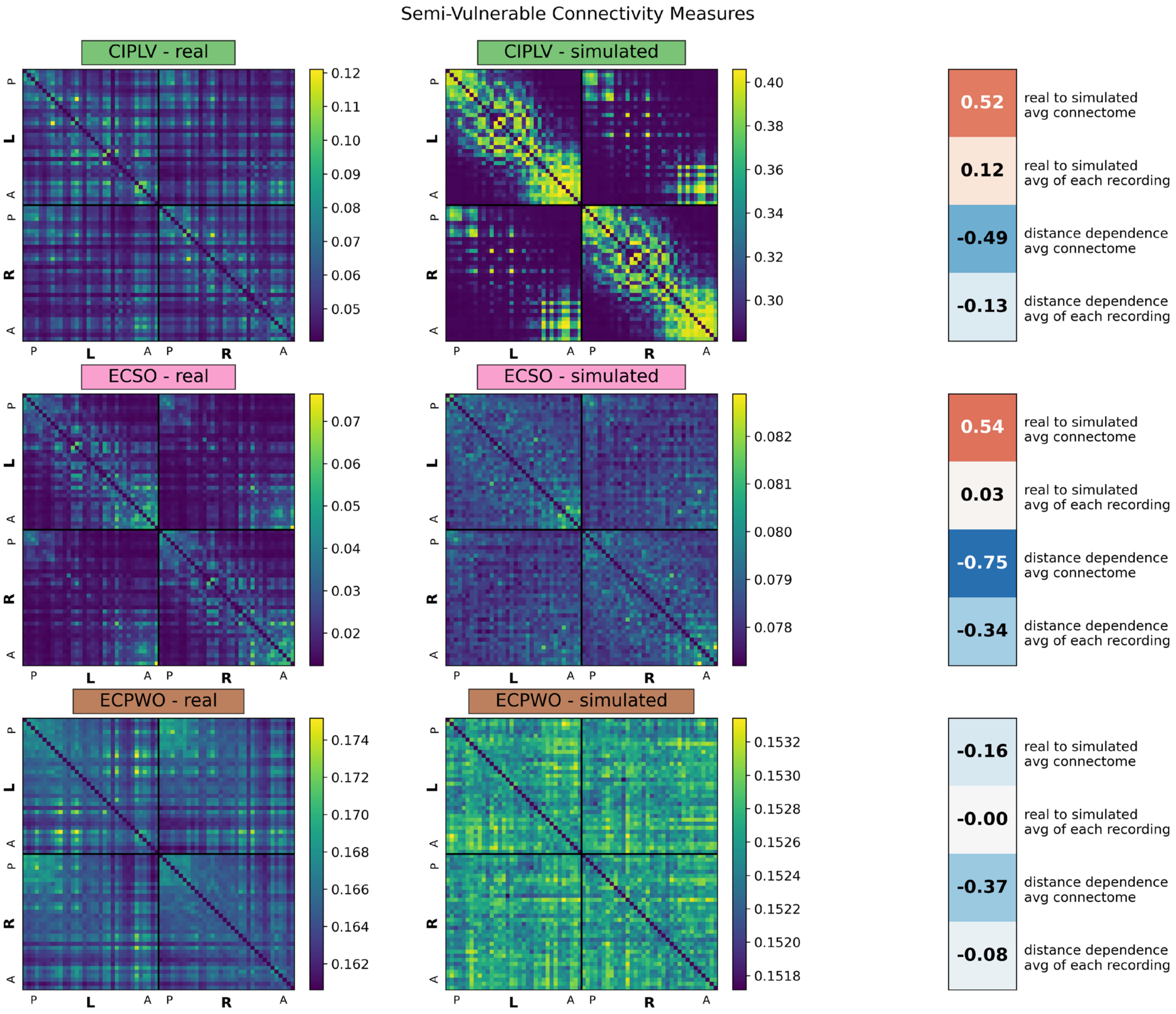


**Figure 2. Average real and simulated alpha band (8-13 Hz) connectomes for connectivity measures semi-vulnerable to volume conduction.** Averages are based off of all 581 recordings across 50 participants for both real and simulated connectomes. Simulated connectomes are derived from random noise projected through the source localization algorithm. Regions were divided by hemisphere (L and R) and arranged anatomically from posterior (P) to anterior (A). Values on the right show, for each connectivity measure, in order: the correlation between the average real and average simulated connectome, the average of the 581 correlations between each real and simulated connectome, the correlation between the average real connectome and average edge length, and the average of the 581 correlations between each real connectome and edge length. *CIPLV: corrected imaginary phase locking value; ECSO: envelope correlation with symmetric orthogonalization; ECPWO: envelope correlation with pairwise orthogonalization.*

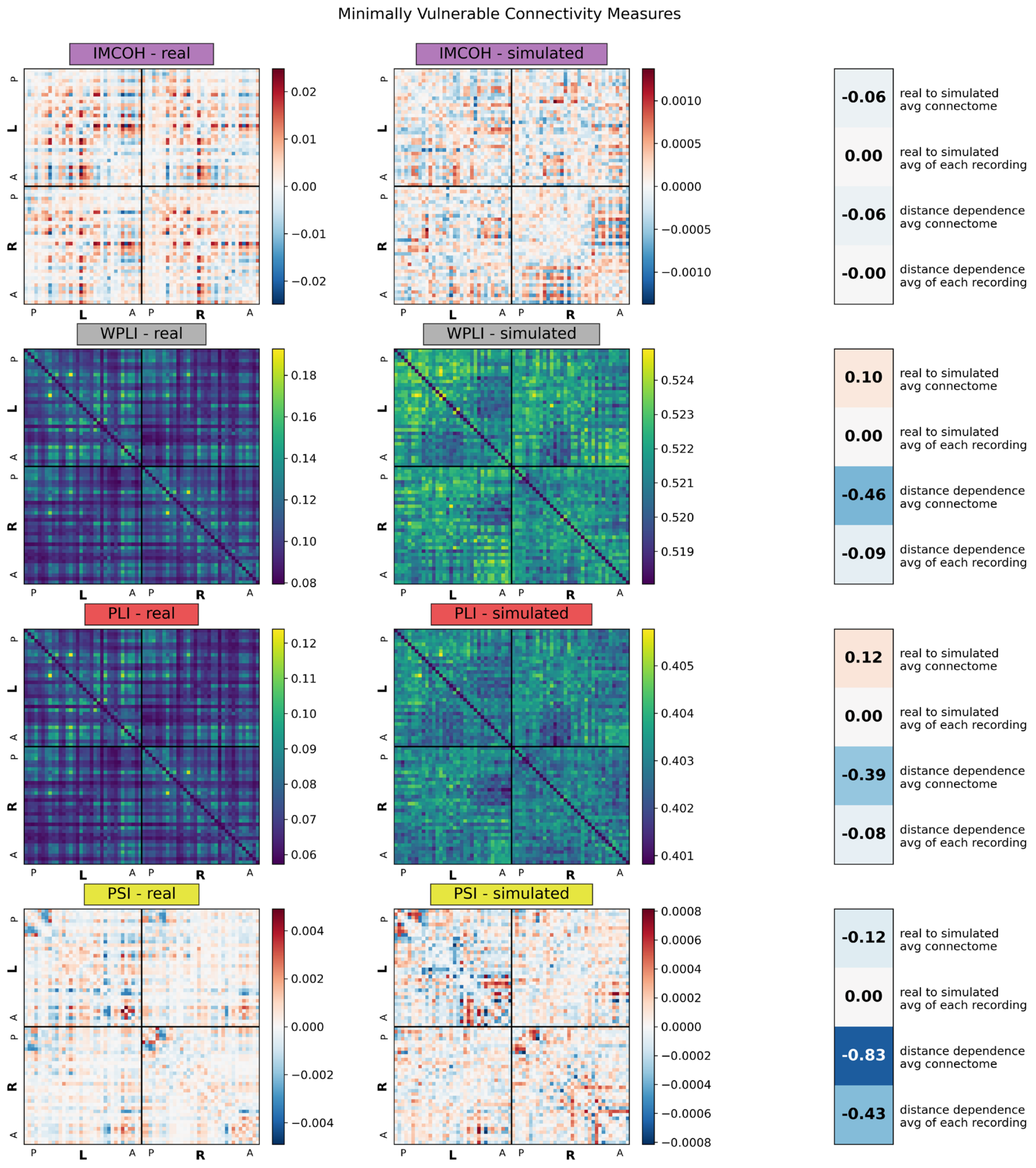


**Figure 3. Average real and simulated alpha band (8-13 Hz) connectomes for connectivity measures minimally vulnerable to volume conduction.** Averages are based off of all 581 recordings across 50 participants for both real and simulated connectomes. Simulated connectomes are derived from random noise projected through the source localization algorithm. Regions were divided by hemisphere (L and R) and arranged anatomically from posterior (P) to anterior (A). Values on the right show, for each connectivity measure, in order: the correlation between the average real and average simulated connectome, the average of the 581 correlations between each real and simulated connectome, the correlation between the average real connectome and average edge length, and the average of the 581 correlations between each real connectome and edge length. *IMCOH: imaginary coherence; WPLI: weighted phase lag index; PLI: phase lag index; PSI: phase slope index.*

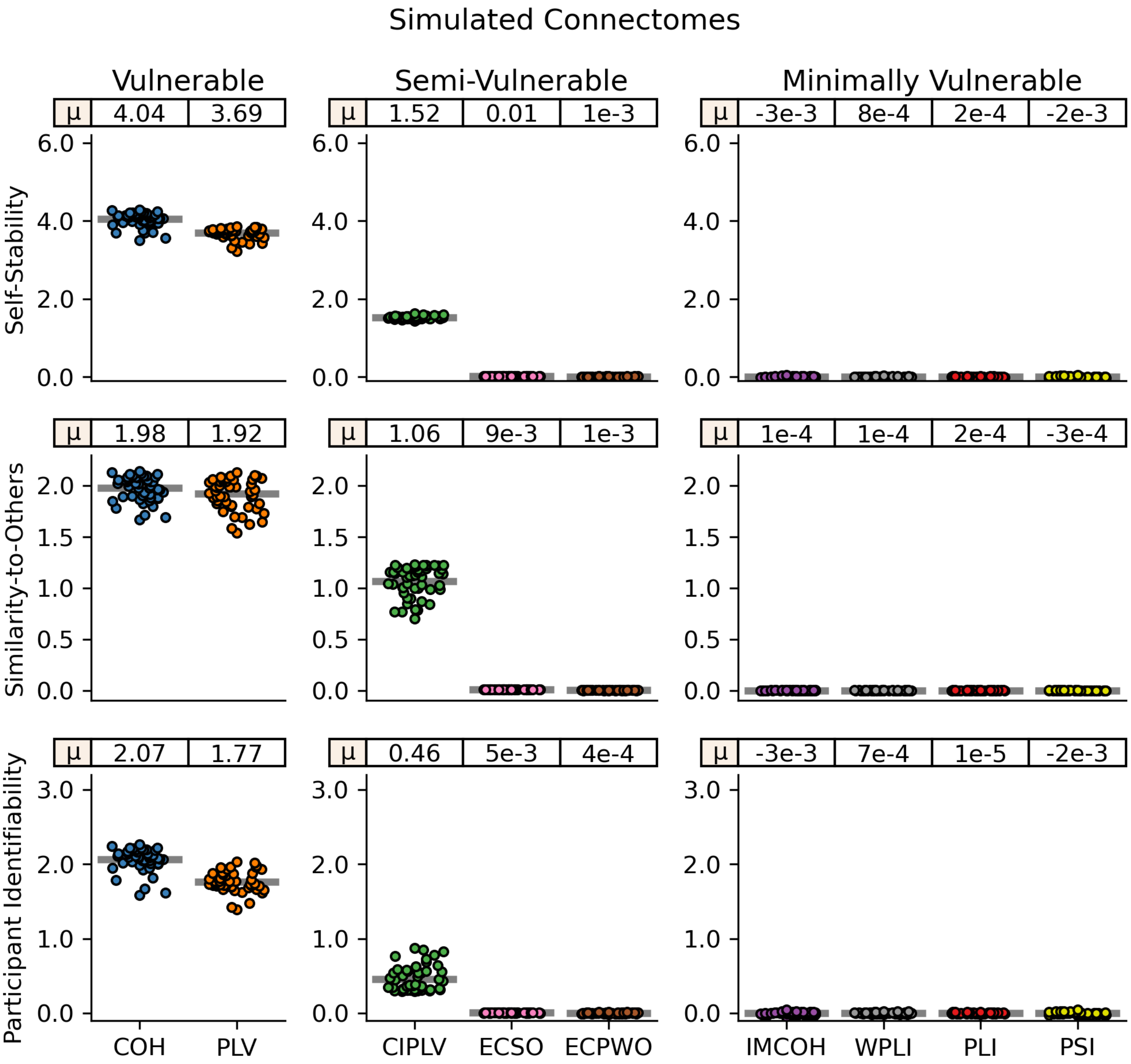


**Figure 4. Alpha band (8-13 Hz) connectome stability and identifiability across FC measures for simulated connectomes.** Simulated connectomes are derived from random noise projected through the source localization algorithm. Each dot represents one participant. Lines represent mean values across participants, which are also displayed at the top of each subplot. From top to bottom, subplots represent: **Mean self-stability**, the average Fisher-z correlation between connectomes of the same participant. **Mean similarity-to-others**, the average Fisher-z correlation between a participant's connectomes and connectomes from all other participants. **Participant identifiability**, the difference between mean self-stability and mean similarity-to-others. Note different y-axes across columns. *COH: coherence; PLV: phase locking value; CIPLV: corrected imaginary phase locking value; ECSO: envelope correlation with symmetric orthogonalization; ECPWO: envelope correlation with pairwise orthogonalization; IMCOH: imaginary coherence; WPLI: weighted phase lag index; PLI: phase lag index; PSI: phase slope index.*

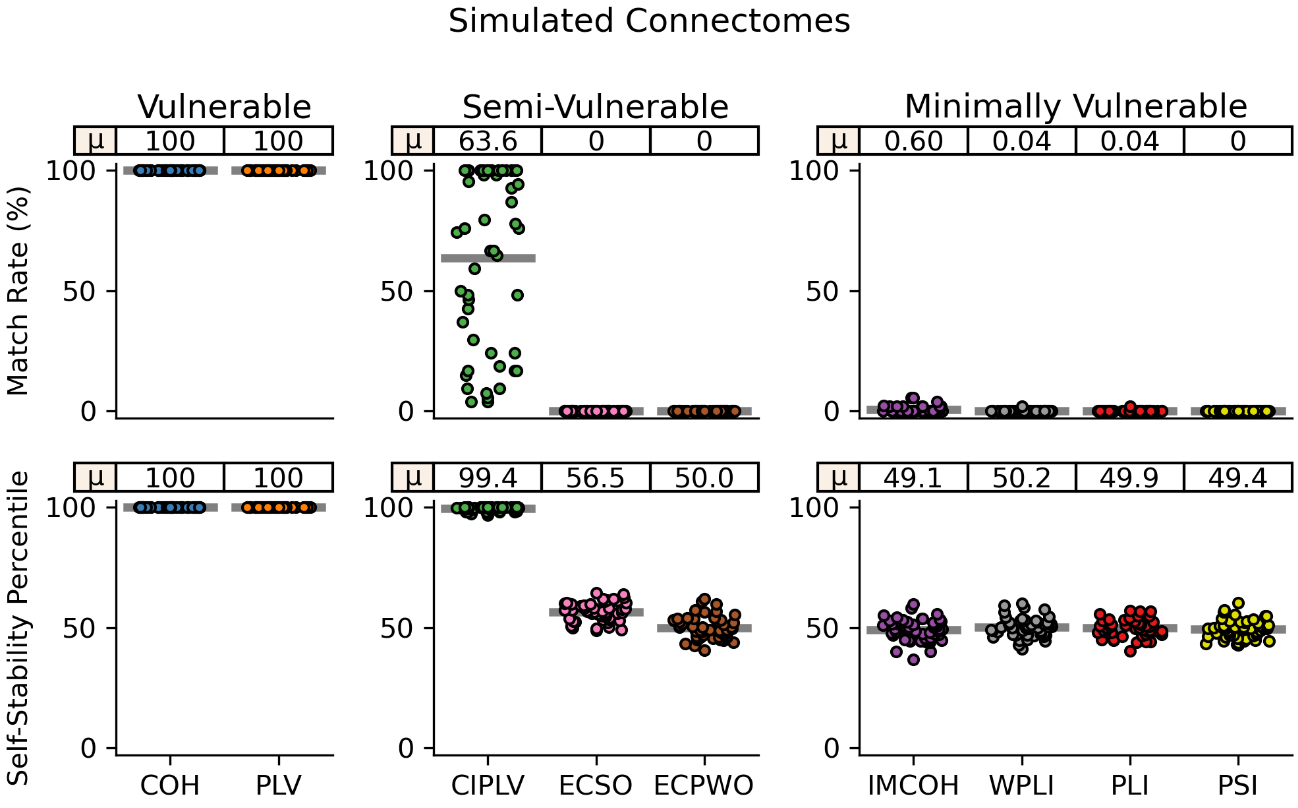


**Supplemental Figure 1. Additional alpha band (8-13 Hz) identifiability metrics across FC measures for simulated connectomes.** Simulated connectomes are derived from random noise projected through the source localization algorithm. Each dot represents one participant. Lines represent mean values across participants, which are also displayed at the top of each subplot. From top to bottom, subplots represent: **Match rate**, the percentage of self-stability scores that were higher than all similarity-to-others scores. **Self-stability percentile**, the average percentile of self-stability scores, relative to similarity-to-others. *COH: coherence; PLV: phase locking value; CIPLV: corrected imaginary phase locking value; ECSO: envelope correlation with symmetric orthogonalization; ECPWO: envelope correlation with pairwise orthogonalization; IMCOH: imaginary coherence; WPLI: weighted phase lag index; PLI: phase lag index; PSI: phase slope index.*

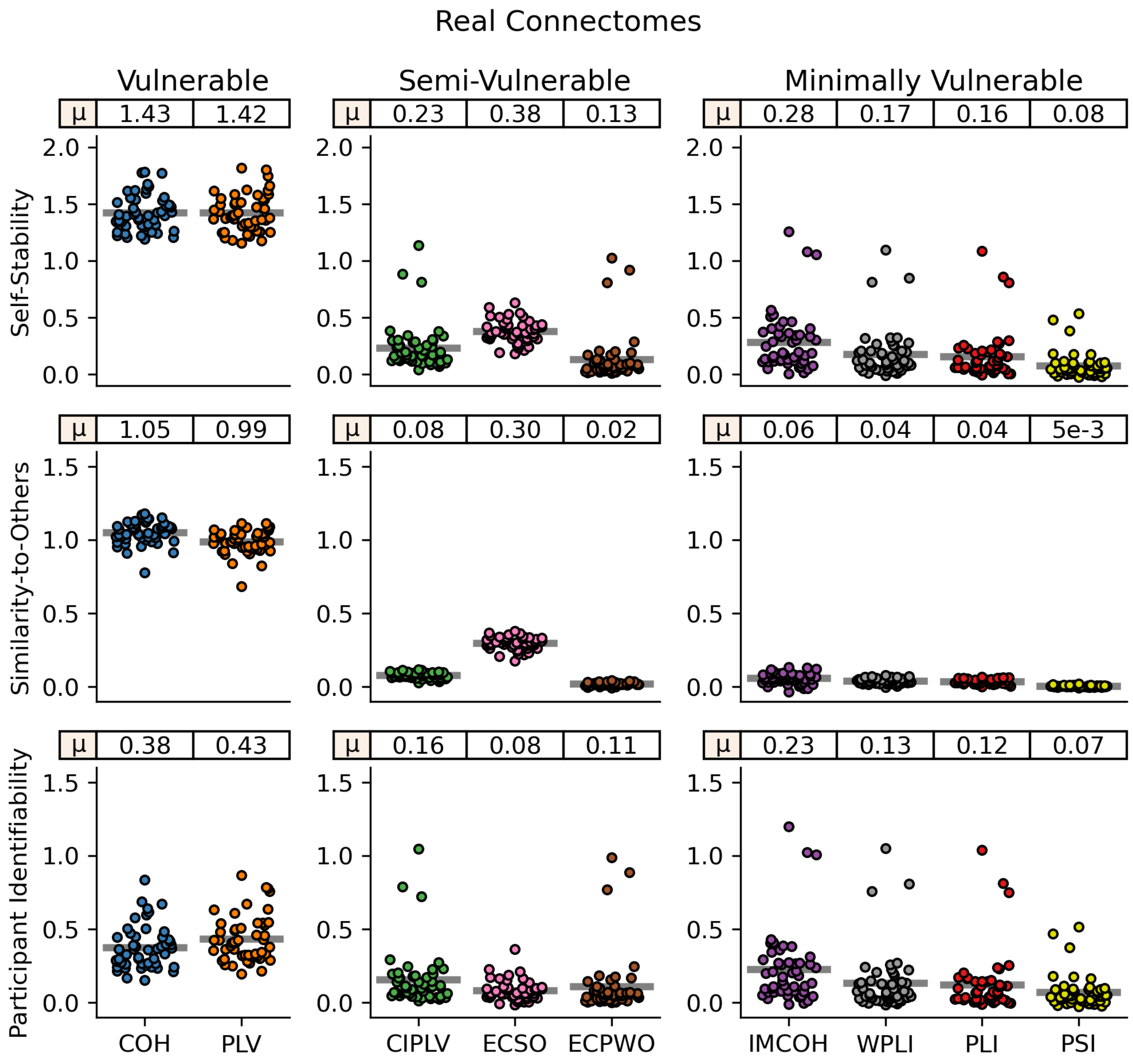


**Figure 5. Alpha band (8-13 Hz) connectome stability and identifiability across FC measures for real connectomes.** Each dot represents one participant. Lines represent mean values across participants, which are also displayed at the top of each subplot. From top to bottom, subplots represent: **Mean self-stability**, the average Fisher-z correlation between connectomes of the same participant, collected on different days. **Mean similarity-to-others**, the average Fisher-z correlation between a participant's connectomes and connectomes from all other participants. **Participant identifiability**, the difference between mean self-stability and mean similarity-to-others. Note different y-axes across columns. *COH: coherence; PLV: phase locking value; CIPLV: corrected imaginary phase locking value; ECSO: envelope correlation with symmetric orthogonalization; ECPWO: envelope correlation with pairwise orthogonalization; IMCOH: imaginary coherence; WPLI: weighted phase lag index; PLI: phase lag index; PSI: phase slope index.*

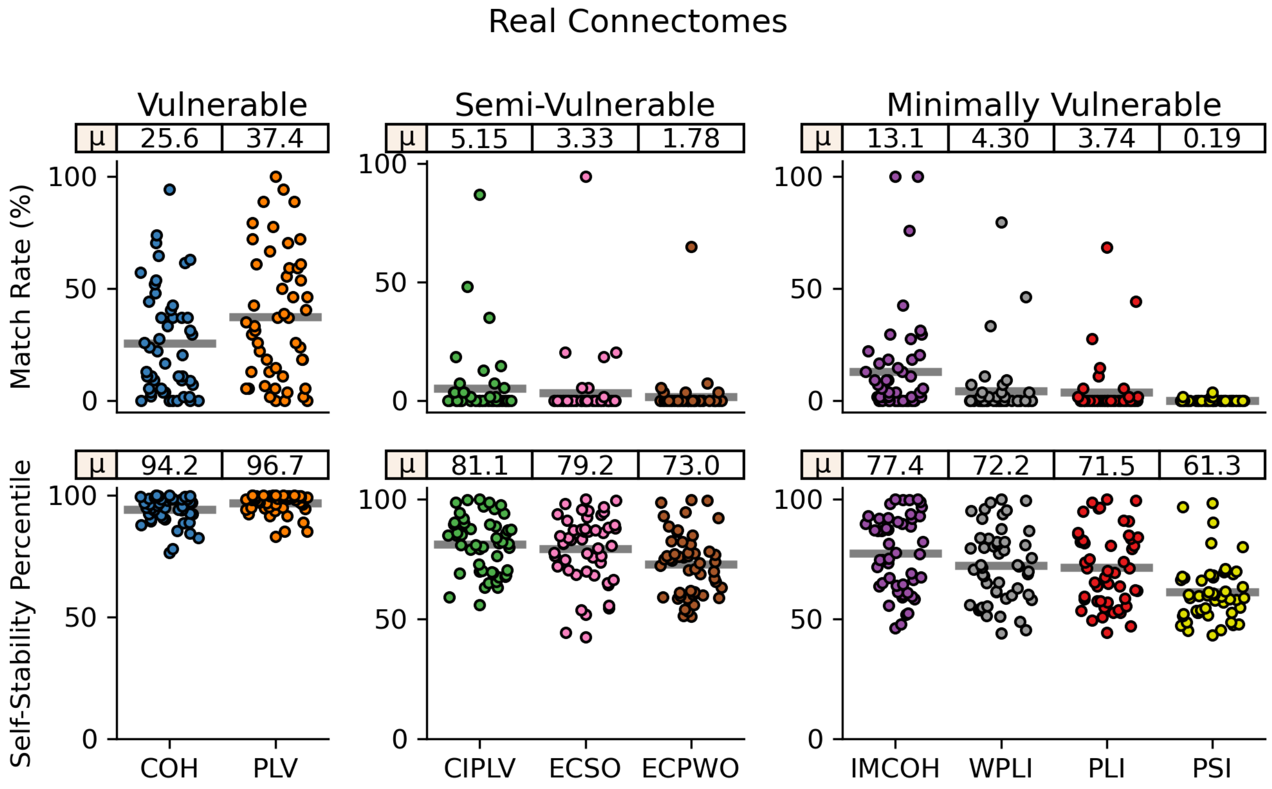


**Supplemental Figure 2. Additional alpha band (8-13 Hz) identifiability metrics across FC measures for real connectomes.** Each dot represents one participant. Lines represent mean values across participants, which are also displayed at the top of each subplot. From top to bottom, subplots represent: **Match rate**, the percentage of self-stability scores that were higher than all similarity-to-others scores. **Self-stability percentile**, the average percentile of self-stability scores, relative to similarity-to-others. *COH: coherence; PLV: phase locking value; CIPLV: corrected imaginary phase locking value; ECSO: envelope correlation with symmetric orthogonalization; ECPWO: envelope correlation with pairwise orthogonalization; IMCOH: imaginary coherence; WPLI: weighted phase lag index; PLI: phase lag index; PSI: phase slope index.*

**Self-Stability**

| | PLV | CIPLV | ECSO | ECPWO | IMCOH | WPLI | PLI | PSI |
|---|---|---|---|---|---|---|---|---|
| COH | p: 0.61 | p: 2.2e-37 | p: 1.0e-38 | p: 1.1e-38 | p: 1.9e-32 | p: 5.9e-38 | p: 4.4e-38 | p: 4.0e-46 |
| PLV | | p: 2.2e-36 | p: 6.3e-38 | p: 5.1e-38 | p: 2.3e-31 | p: 6.7e-37 | p: 4.8e-37 | p: 7.2e-45 |
| CIPLV | | | p: 8.5e-7 | p: 2.0e-8 | p: 2.8e-4 | p: 8.3e-23 | p: 6.5e-28 | p: 3.8e-12 |
| ECSO | | | | p: 9.2e-13 | p: 6.6e-3 | p: 1.4e-9 | p: 1.7e-10 | p: 5.4e-23 |
| ECPWO | | | | | p: 6.0e-9 | p: 6.9e-3 | p: 0.053 | p: 6.3e-3 |
| IMCOH | | | | | | p: 8.7e-14 | p: 2.6e-14 | p: 1.7e-10 |
| WPLI | | | | | | | p: 2.2e-6 | p: 3.0e-6 |
| PLI | | | | | | | | p: 3.6e-5 |

**Similarity-to-Others**

| | PLV | CIPLV | ECSO | ECPWO | IMCOH | WPLI | PLI | PSI |
|---|---|---|---|---|---|---|---|---|
| COH | p: 3.0e-29 | p: 2.0e-56 | p: 5.9e-48 | p: 3.2e-57 | p: 1.3e-55 | p: 4.5e-57 | p: 8.9e-57 | p: 1.0e-57 |
| PLV | | p: 9.8e-54 | p: 5.4e-45 | p: 7.9e-55 | p: 7.4e-53 | p: 2.8e-54 | p: 4.2e-54 | p: 2.6e-55 |
| CIPLV | | | p: 4.5e-38 | p: 2.4e-25 | p: 2.2e-7 | p: 4.9e-35 | p: 2.5e-39 | p: 3.2e-30 |
| ECSO | | | | p: 1.1e-41 | p: 3.1e-35 | p: 1.1e-40 | p: 1.3e-41 | p: 3.1e-43 |
| ECPWO | | | | | p: 1.1e-8 | p: 2.6e-9 | p: 3.8e-8 | p: 1.4e-14 |
| IMCOH | | | | | | p: 1.5e-5 | p: 4.8e-7 | p: 3.8e-13 |
| WPLI | | | | | | | p: 7.9e-9 | p: 1.0e-17 |
| PLI | | | | | | | | p: 2.0e-17 |

**Participant Identifiability**

| | PLV | CIPLV | ECSO | ECPWO | IMCOH | WPLI | PLI | PSI |
|---|---|---|---|---|---|---|---|---|
| COH | p: 6.4e-18 | p: 4.7e-11 | p: 5.0e-20 | p: 7.4e-14 | p: 4.8e-5 | p: 8.1e-12 | p: 2.2e-12 | p: 7.6e-20 |
| PLV | | p: 9.5e-14 | p: 3.5e-22 | p: 1.8e-16 | p: 2.4e-7 | p: 2.5e-14 | p: 8.2e-15 | p: 1.1e-21 |
| CIPLV | | | p: 2.7e-3 | p: 2.1e-3 | p: 1.2e-7 | p: 2.0e-8 | p: 3.0e-14 | p: 3.9e-6 |
| ECSO | | | | p: 0.24 | p: 2.3e-5 | p: 0.045 | p: 0.11 | p: 0.44 |
| ECPWO | | | | | p: 1.0e-7 | p: 0.099 | p: 0.36 | p: 0.040 |
| IMCOH | | | | | | p: 5.2e-13 | p: 1.9e-13 | p: 3.5e-8 |
| WPLI | | | | | | | p: 7.0e-5 | p: 8.6e-4 |
| PLI | | | | | | | | p: 4.7e-3 |

**Match Rate (%)**

| | PLV | CIPLV | ECSO | ECPWO | IMCOH | WPLI | PLI | PSI |
|---|---|---|---|---|---|---|---|---|
| COH | p: 1.8e-9 | p: 4.7e-7 | p: 1.7e-7 | p: 1.6e-8 | p: 5.3e-3 | p: 1.3e-7 | p: 6.8e-8 | p: 1.1e-9 |
| PLV | | p: 4.0e-10 | p: 3.9e-10 | p: 3.9e-11 | p: 6.3e-6 | p: 1.4e-10 | p: 9.7e-11 | p: 3.2e-12 |
| CIPLV | | | p: 0.32 | p: 0.072 | p: 3.3e-5 | p: 0.050 | p: 7.1e-3 | p: 0.018 |
| ECSO | | | | p: 0.072 | p: 4.8e-4 | p: 0.57 | p: 0.79 | p: 0.10 |
| ECPWO | | | | | p: 2.8e-4 | p: 0.14 | p: 0.18 | p: 0.20 |
| IMCOH | | | | | | p: 1.7e-5 | p: 1.9e-5 | p: 1.6e-4 |
| WPLI | | | | | | | p: 0.10 | p: 0.031 |
| PLI | | | | | | | | p: 0.035 |

**Self-Stability Percentile**

| | PLV | CIPLV | ECSO | ECPWO | IMCOH | WPLI | PLI | PSI |
|---|---|---|---|---|---|---|---|---|
| COH | p: 4.3e-11 | p: 5.8e-11 | p: 5.1e-10 | p: 1.4e-15 | p: 2.4e-9 | p: 5.8e-14 | p: 8.6e-14 | p: 2.9e-24 |
| PLV | | p: 1.8e-13 | p: 3.1e-12 | p: 1.7e-17 | p: 4.4e-11 | p: 1.4e-15 | p: 2.1e-15 | p: 8.7e-26 |
| CIPLV | | | p: 0.34 | p: 2.5e-4 | p: 8.1e-3 | p: 3.5e-13 | p: 1.2e-12 | p: 1.7e-15 |
| ECSO | | | | p: 7.8e-3 | p: 0.48 | p: 6.1e-3 | p: 3.3e-3 | p: 1.4e-10 |
| ECPWO | | | | | p: 0.059 | p: 0.74 | p: 0.53 | p: 3.1e-8 |
| IMCOH | | | | | | p: 7.6e-6 | p: 1.7e-6 | p: 4.4e-10 |
| WPLI | | | | | | | p: 0.18 | p: 3.9e-7 |
| PLI | | | | | | | | p: 1.5e-6 |

**Supplemental Figure 3. Statistical comparisons for alpha band (8-13 Hz) connectivity between self-stability and identifiability measures, as presented in Figure 5 and Supplemental Figure 2.** Between each FC measure, a paired t-test was used to assess the statistical difference between participants' scores. Yellow shading = $p < 0.05$ Bonferroni corrected; green shading = $p < 0.05$ uncorrected. *COH: coherence; PLV: phase locking value; CIPLV: corrected imaginary phase locking value; ECSO: envelope correlation with symmetric orthogonalization; ECPWO: envelope correlation with pairwise orthogonalization; IMCOH: imaginary coherence; WPLI: weighted phase lag index; PLI: phase lag index; PSI: phase slope index.*

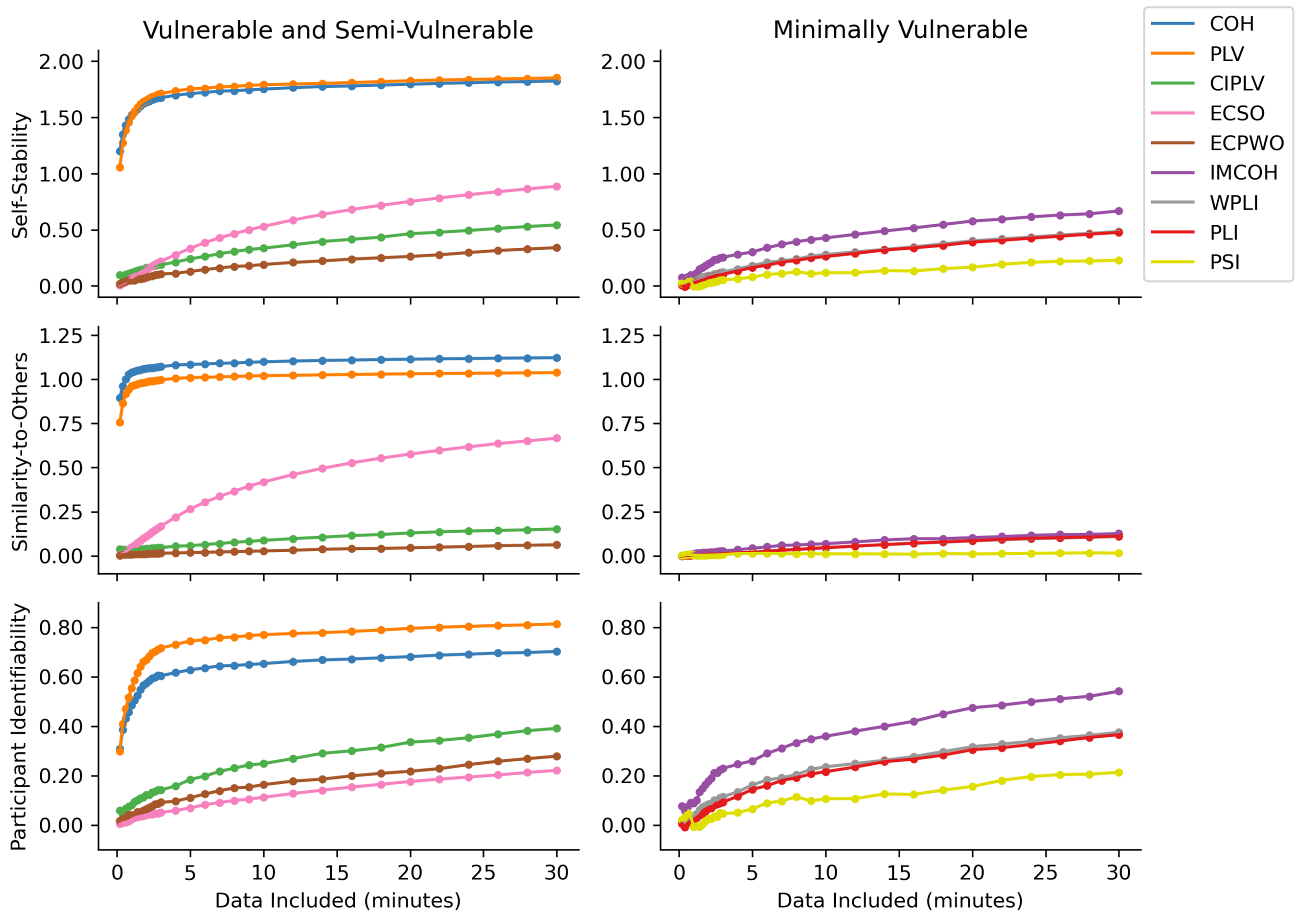


**Figure 6. Effect of different recording lengths on self-stability and identifiability, across FC measures, for alpha band (8-13 Hz) connectivity.** Points shown are across-participant averages. For each participant, the recordings from their first two sessions and from their last two sessions were concatenated, giving two sets of data per participant. Data included refers to the total amount of data in each set, e.g., "12 minutes included" would refer to using the first 2 minutes available from each recording, 6 recordings in the first two sessions and 6 in the last two sessions. From top to bottom, subplots represent: **Mean self-stability**, the average Fisher-z correlation between connectomes of the same participant, averaged across participants. **Mean similarity-to-others**, the average Fisher-z correlation between a participant's connectomes and connectomes from all other participants, averaged across participants. **Mean participant identifiability**, the difference between self-stability and mean similarity-to-others, averaged across participants. *COH: coherence; PLV: phase locking value; CIPLV: corrected imaginary phase locking value; ECSO: envelope correlation with symmetric orthogonalization; ECPWO: envelope correlation with pairwise orthogonalization; IMCOH: imaginary coherence; WPLI: weighted phase lag index; PLI: phase lag index; PSI: phase slope index.*

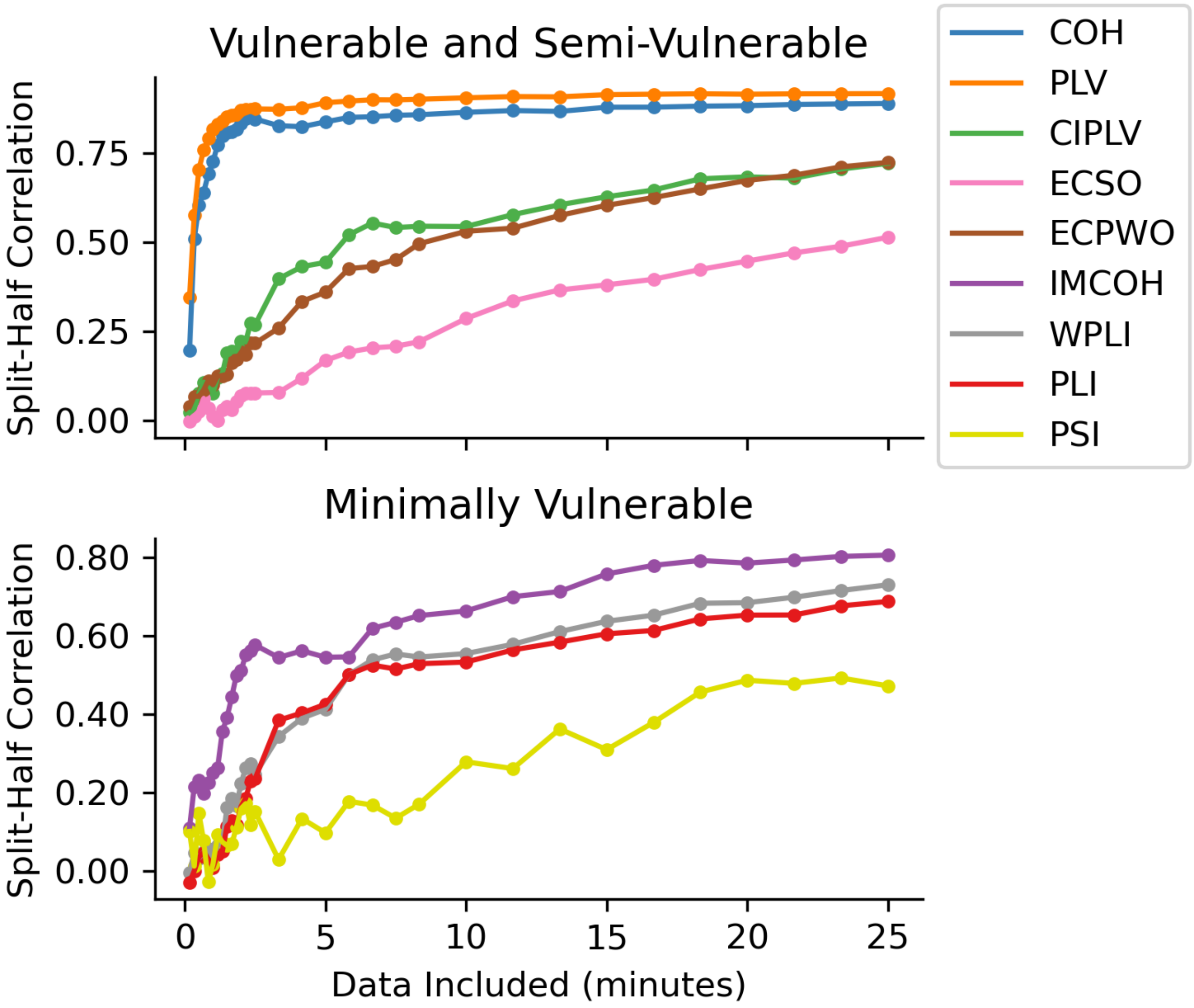


**Figure 7. Effect of different recording lengths on the stability of age group effects across recording sessions for alpha band (8-13 Hz) connectivity.** For each participant, the recordings from their first two sessions and from their last two sessions were concatenated, giving two sets of data per participant, from which connectomes were generated. Across parent-child pairs, using each participant's first set of data, paired t-tests were used to calculate t values of age group effects for each connectivity edge. This was repeated for the second set of data, and the two sets of t values were then correlated, to assess the stability of measured age group effects. Data included refers to the total amount of data in each set, e.g., "10 minutes included" would refer to using the first 2 minutes available from each recording, 5 recordings in the first two sessions and 5 in the last two sessions. *COH: coherence; PLV: phase locking value; CIPLV: corrected imaginary phase locking value; ECSO: envelope correlation with symmetric orthogonalization; ECPWO: envelope correlation with pairwise orthogonalization; IMCOH: imaginary coherence; WPLI: weighted phase lag index; PLI: phase lag index; PSI: phase slope index.*

**Beta Band Results**

In the main body of the paper, results were presented for broadband connectivity. Here, results are presented for **beta band connectivity (13.0 – 30.0 Hz)**. For ease of comparison, figures are labelled as either 'Figure' or 'Supplemental Figure' to match their designation elsewhere.

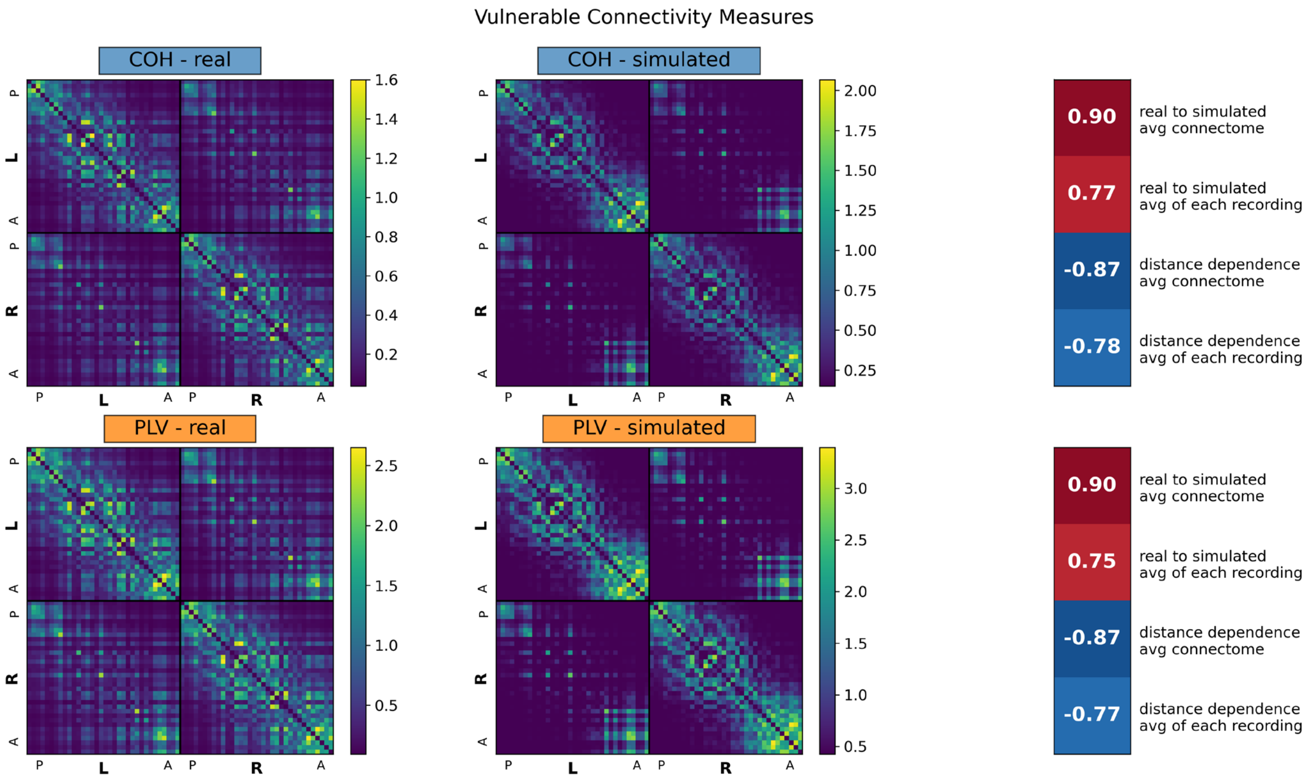


**Figure 1. Average real and simulated beta band (13-30 Hz) connectomes for connectivity measures vulnerable to volume conduction.** Averages are based off of all 581 recordings across 50 participants for both real and simulated connectomes. Simulated connectomes are derived from random noise projected through the source localization algorithm. Regions were divided by hemisphere (L and R) and arranged anatomically from posterior (P) to anterior (A). Values on the right show, for each connectivity measure, in order: the correlation between the average real and average simulated connectome, the average of the 581 correlations between each real and simulated connectome, the correlation between the average real connectome and average edge length, and the average of the 581 correlations between each real connectome and edge length. *COH: coherence; PLV: phase locking value.*

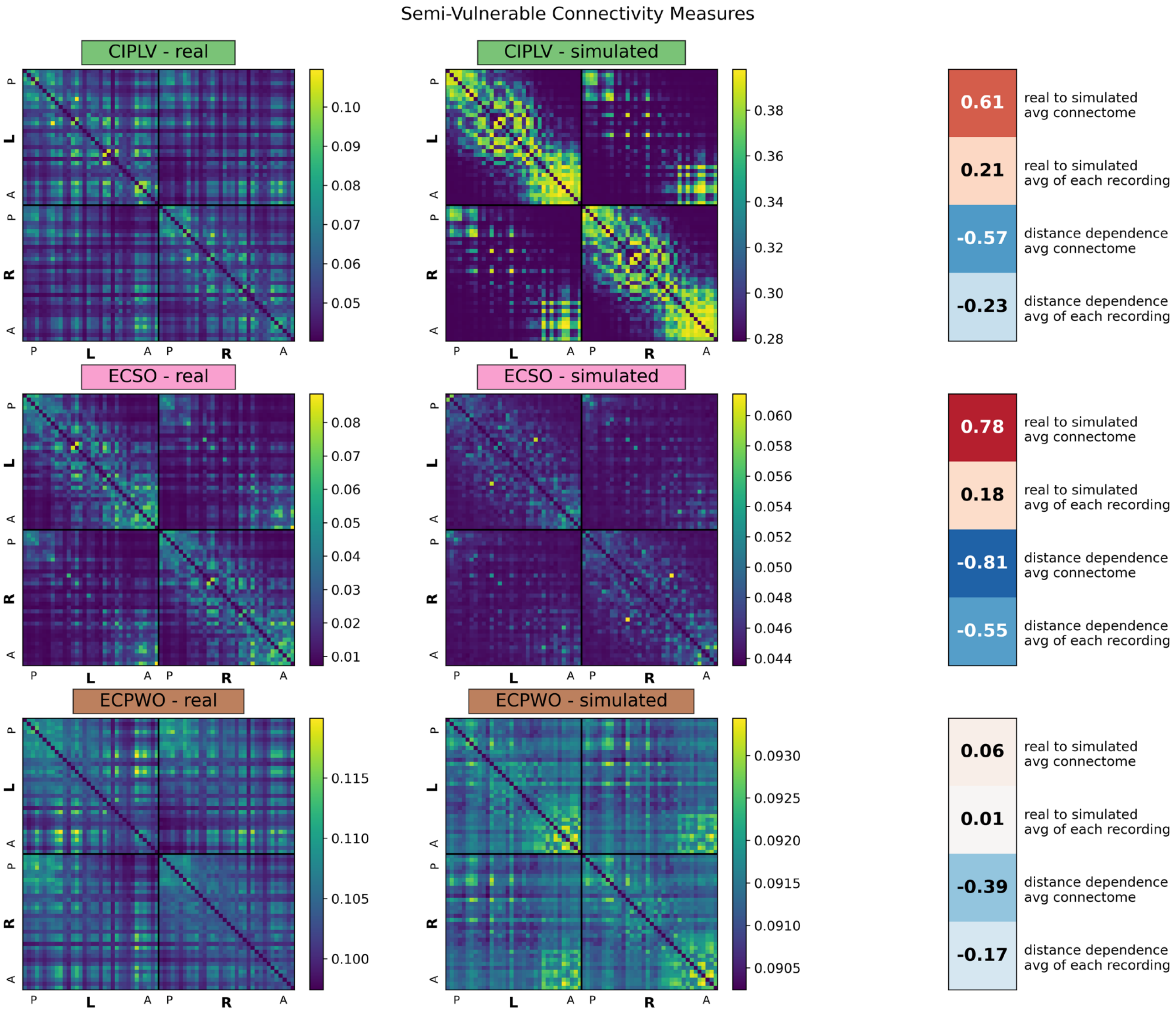


**Figure 2. Average real and simulated beta band (13-30 Hz) connectomes for connectivity measures semi-vulnerable to volume conduction.** Averages are based off of all 581 recordings across 50 participants for both real and simulated connectomes. Simulated connectomes are derived from random noise projected through the source localization algorithm. Regions were divided by hemisphere (L and R) and arranged anatomically from posterior (P) to anterior (A). Values on the right show, for each connectivity measure, in order: the correlation between the average real and average simulated connectome, the average of the 581 correlations between each real and simulated connectome, the correlation between the average real connectome and average edge length, and the average of the 581 correlations between each real connectome and edge length. *CIPLV: corrected imaginary phase locking value; ECSO: envelope correlation with symmetric orthogonalization; ECPWO: envelope correlation with pairwise orthogonalization.*

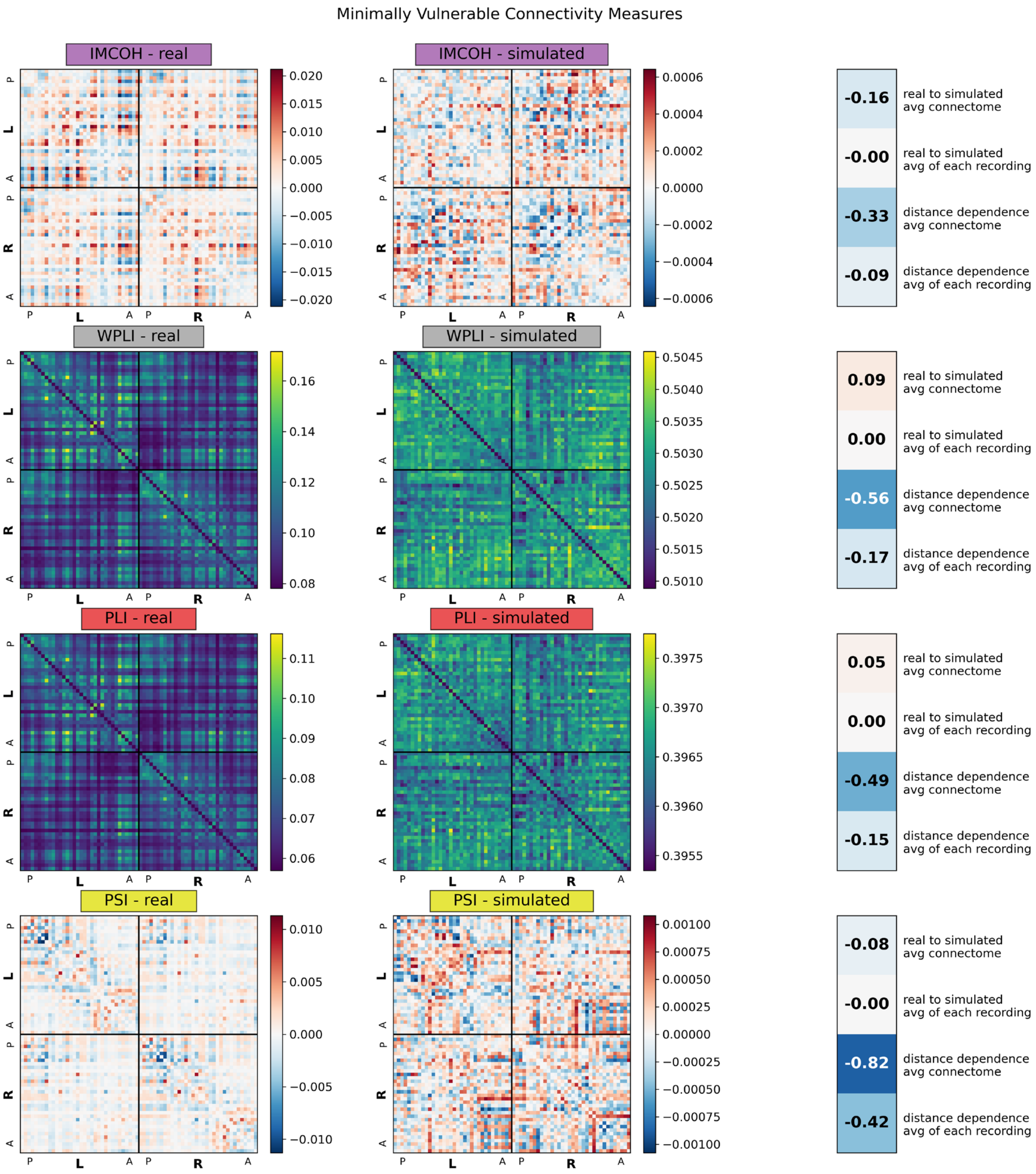


**Figure 3. Average real and simulated beta band (13-30 Hz) connectomes for connectivity measures minimally vulnerable to volume conduction.** Averages are based off of all 581 recordings across 50 participants for both real and simulated connectomes. Simulated connectomes are derived from random noise projected through the source localization algorithm. Regions were divided by hemisphere (L and R) and arranged anatomically from posterior (P) to anterior (A). Values on the right show, for each connectivity measure, in order: the correlation between the average real and average simulated connectome, the average of the 581 correlations between each real and simulated connectome, the correlation between the average real connectome and average edge length, and the average of the 581 correlations between each real connectome and edge length. *IMCOH: imaginary coherence; WPLI: weighted phase lag index; PLI: phase lag index; PSI: phase slope index.*

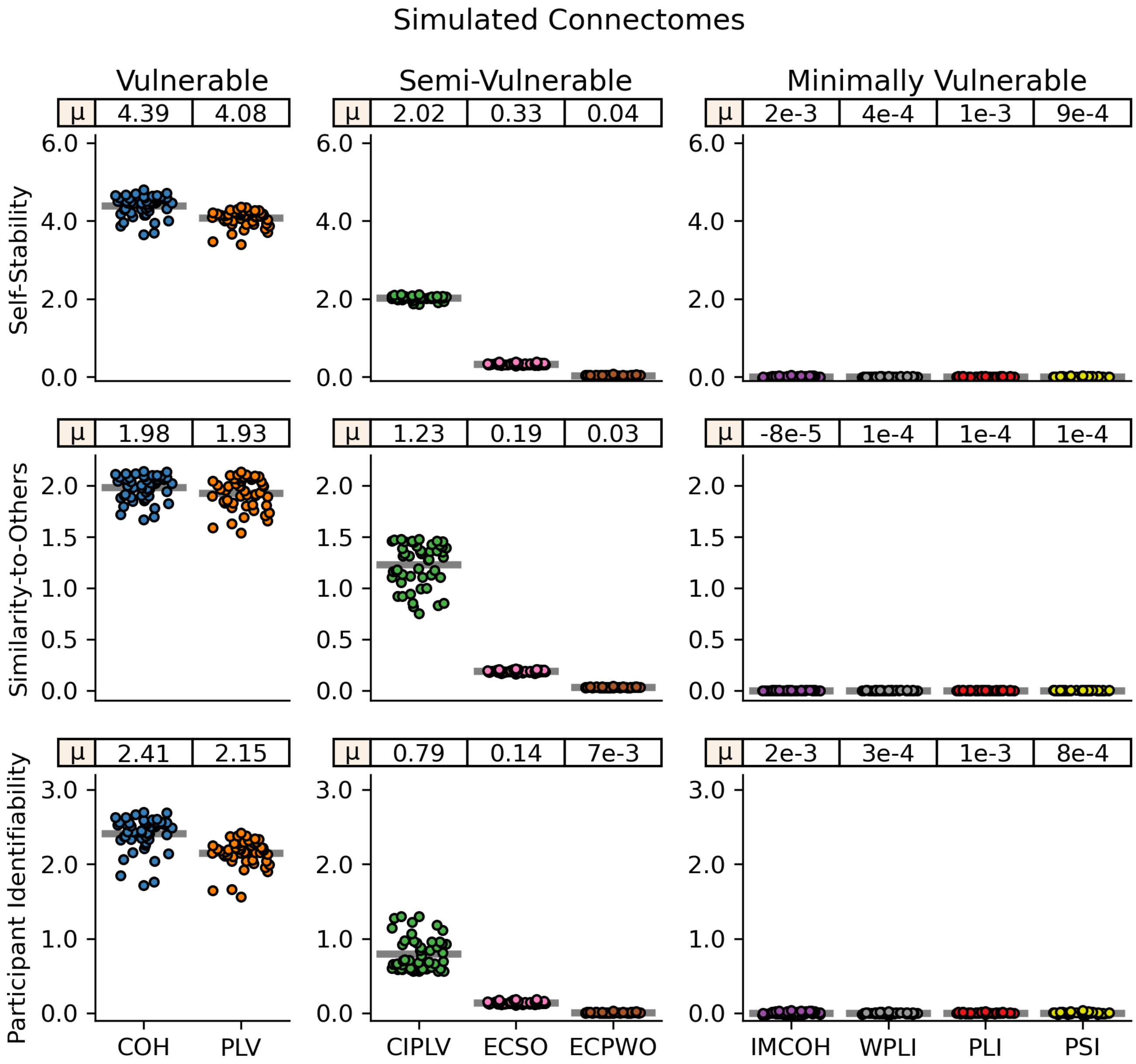


**Figure 4. Beta band (13-30 Hz) connectome stability and identifiability across FC measures for simulated connectomes.** Simulated connectomes are derived from random noise projected through the source localization algorithm. Each dot represents one participant. Lines represent mean values across participants, which are also displayed at the top of each subplot. From top to bottom, subplots represent: **Mean self-stability**, the average Fisher-z correlation between connectomes of the same participant. **Mean similarity-to-others**, the average Fisher-z correlation between a participant's connectomes and connectomes from all other participants. **Participant identifiability**, the difference between mean self-stability and mean similarity-to-others. Note different y-axes across columns. *COH: coherence; PLV: phase locking value; CIPLV: corrected imaginary phase locking value; ECSO: envelope correlation with symmetric orthogonalization; ECPWO: envelope correlation with pairwise orthogonalization; IMCOH: imaginary coherence; WPLI: weighted phase lag index; PLI: phase lag index; PSI: phase slope index.*

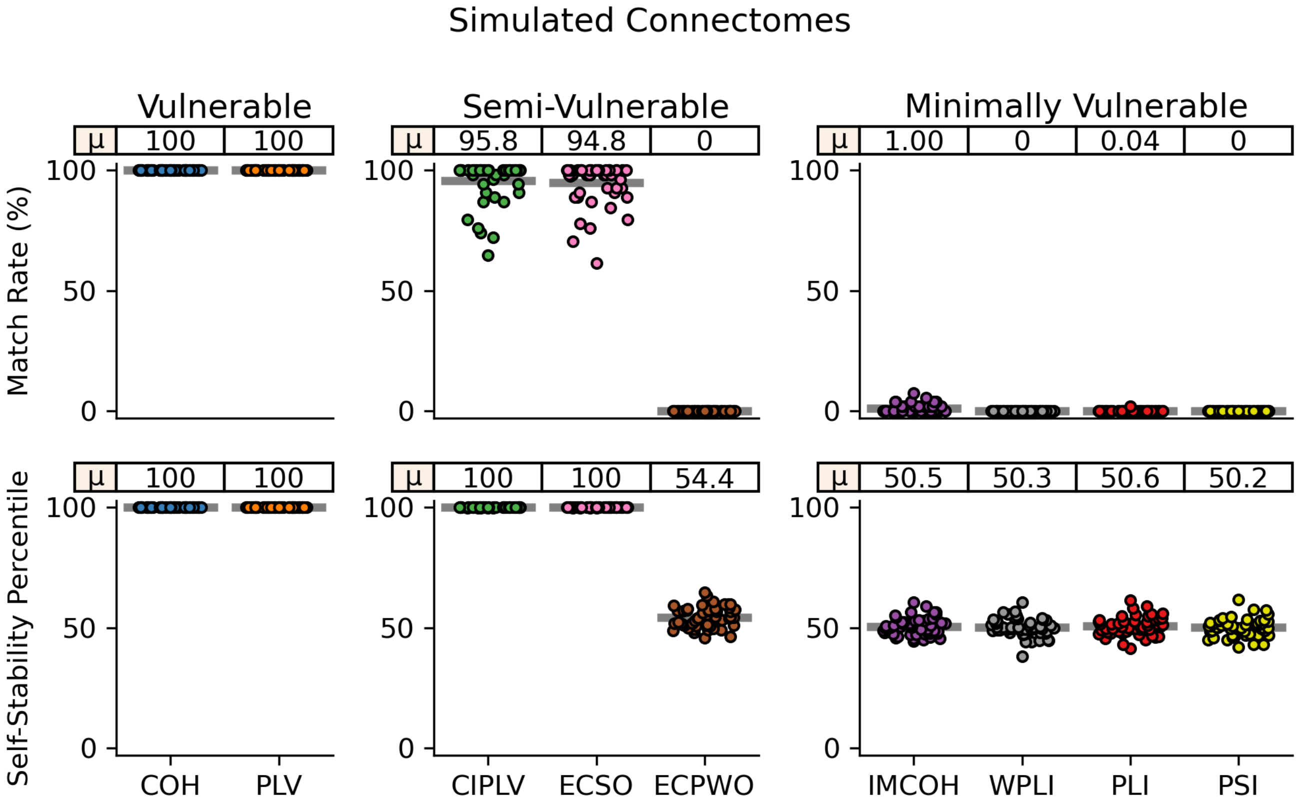


**Supplemental Figure 1. Additional beta band (13-30 Hz) identifiability metrics across FC measures for simulated connectomes.** Simulated connectomes are derived from random noise projected through the source localization algorithm. Each dot represents one participant. Lines represent mean values across participants, which are also displayed at the top of each subplot. From top to bottom, subplots represent: **Match rate**, the percentage of self-stability scores that were higher than all similarity-to-others scores. **Self-stability percentile**, the average percentile of self-stability scores, relative to similarity-to-others. *COH: coherence; PLV: phase locking value; CIPLV: corrected imaginary phase locking value; ECSO: envelope correlation with symmetric orthogonalization; ECPWO: envelope correlation with pairwise orthogonalization; IMCOH: imaginary coherence; WPLI: weighted phase lag index; PLI: phase lag index; PSI: phase slope index.*

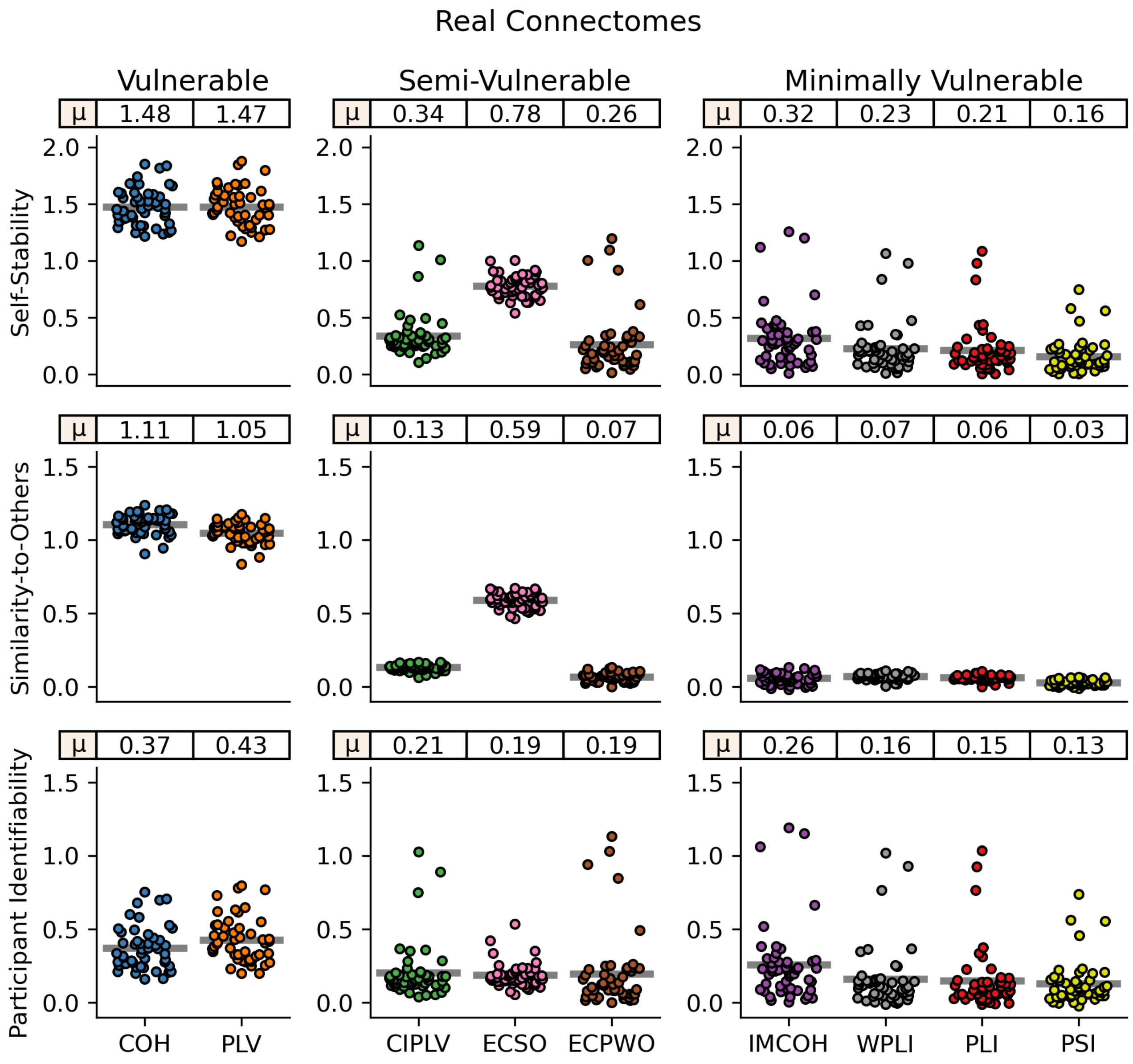


**Figure 5. Beta band (13-30 Hz) connectome stability and identifiability across FC measures for real connectomes.** Each dot represents one participant. Lines represent mean values across participants, which are also displayed at the top of each subplot. From top to bottom, subplots represent: **Mean self-stability**, the average Fisher-z correlation between connectomes of the same participant, collected on different days. **Mean similarity-to-others**, the average Fisher-z correlation between a participant's connectomes and connectomes from all other participants. **Participant identifiability**, the difference between mean self-stability and mean similarity-to-others. Note different y-axes across columns. *COH: coherence; PLV: phase locking value; CIPLV: corrected imaginary phase locking value; ECSO: envelope correlation with symmetric orthogonalization; ECPWO: envelope correlation with pairwise orthogonalization; IMCOH: imaginary coherence; WPLI: weighted phase lag index; PLI: phase lag index; PSI: phase slope index.*

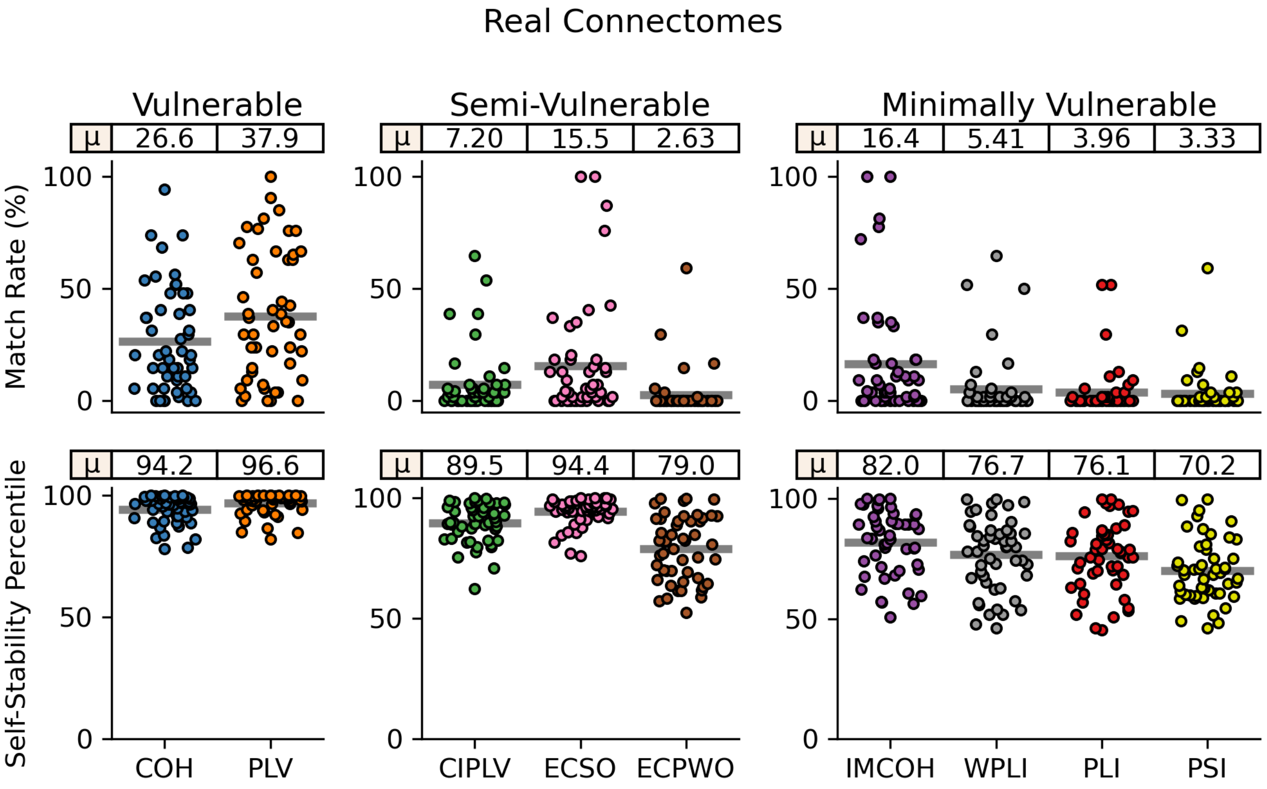


**Supplemental Figure 2. Additional beta band (13-30 Hz) identifiability metrics across FC measures for real connectomes.** Each dot represents one participant. Lines represent mean values across participants, which are also displayed at the top of each subplot. From top to bottom, subplots represent: **Match rate**, the percentage of self-stability scores that were higher than all similarity-to-others scores. **Self-stability percentile**, the average percentile of self-stability scores, relative to similarity-to-others. *COH: coherence; PLV: phase locking value; CIPLV: corrected imaginary phase locking value; ECSO: envelope correlation with symmetric orthogonalization; ECPWO: envelope correlation with pairwise orthogonalization; IMCOH: imaginary coherence; WPLI: weighted phase lag index; PLI: phase lag index; PSI: phase slope index.*

**Self-Stability**

| | PLV | CIPLV | ECSO | ECPWO | IMCOH | WPLI | PLI | PSI |
|---|---|---|---|---|---|---|---|---|
| COH | p: 0.41 | p: 1.8e-37 | p: 1.7e-34 | p: 5.3e-35 | p: 1.8e-32 | p: 9.7e-38 | p: 1.7e-37 | p: 1.1e-42 |
| PLV | | p: 4.2e-37 | p: 2.1e-33 | p: 1.5e-34 | p: 5.1e-32 | p: 2.7e-37 | p: 4.3e-37 | p: 1.3e-41 |
| CIPLV | | | p: 7.4e-23 | p: 7.7e-4 | p: 0.17 | p: 2.3e-25 | p: 9.4e-29 | p: 2.6e-13 |
| ECSO | | | | p: 4.5e-20 | p: 1.6e-17 | p: 1.5e-24 | p: 6.0e-25 | p: 3.4e-35 |
| ECPWO | | | | | p: 7.0e-3 | p: 0.10 | p: 0.015 | p: 1.2e-4 |
| IMCOH | | | | | | p: 6.5e-9 | p: 3.4e-11 | p: 2.3e-7 |
| WPLI | | | | | | | p: 2.3e-6 | p: 7.2e-4 |
| PLI | | | | | | | | p: 9.1e-3 |

**Similarity-to-Others**

| | PLV | CIPLV | ECSO | ECPWO | IMCOH | WPLI | PLI | PSI |
|---|---|---|---|---|---|---|---|---|
| COH | p: 1.3e-36 | p: 7.4e-61 | p: 8.0e-45 | p: 2.1e-59 | p: 4.3e-59 | p: 2.0e-60 | p: 4.5e-60 | p: 7.6e-62 |
| PLV | | p: 1.2e-58 | p: 2.6e-41 | p: 1.5e-57 | p: 6.1e-57 | p: 4.8e-58 | p: 9.0e-58 | p: 1.2e-59 |
| CIPLV | | | p: 9.0e-49 | p: 1.7e-19 | p: 4.5e-22 | p: 6.9e-39 | p: 6.2e-41 | p: 7.0e-32 |
| ECSO | | | | p: 1.6e-49 | p: 1.6e-46 | p: 4.5e-51 | p: 2.9e-51 | p: 9.4e-55 |
| ECPWO | | | | | p: 0.11 | p: 0.72 | p: 0.29 | p: 4.9e-11 |
| IMCOH | | | | | | p: 0.012 | p: 0.28 | p: 7.8e-6 |
| WPLI | | | | | | | p: 1.5e-12 | p: 7.8e-15 |
| PLI | | | | | | | | p: 2.2e-12 |

**Participant Identifiability**

| | PLV | CIPLV | ECSO | ECPWO | IMCOH | WPLI | PLI | PSI |
|---|---|---|---|---|---|---|---|---|
| COH | p: 1.3e-21 | p: 1.8e-7 | p: 4.2e-15 | p: 1.5e-6 | p: 2.4e-3 | p: 4.3e-9 | p: 1.9e-9 | p: 8.7e-14 |
| PLV | | p: 1.4e-10 | p: 2.4e-18 | p: 4.4e-9 | p: 2.0e-5 | p: 7.9e-12 | p: 3.8e-12 | p: 2.5e-16 |
| CIPLV | | | p: 0.37 | p: 0.57 | p: 3.7e-4 | p: 6.8e-12 | p: 1.3e-15 | p: 2.8e-5 |
| ECSO | | | | p: 0.77 | p: 0.021 | p: 0.26 | p: 0.12 | p: 2.4e-4 |
| ECPWO | | | | | p: 5.1e-4 | p: 0.056 | p: 0.013 | p: 5.1e-3 |
| IMCOH | | | | | | p: 9.1e-12 | p: 1.5e-13 | p: 1.1e-6 |
| WPLI | | | | | | | p: 3.8e-4 | p: 0.098 |
| PLI | | | | | | | | p: 0.28 |

**Match Rate (%)**

| | PLV | CIPLV | ECSO | ECPWO | IMCOH | WPLI | PLI | PSI |
|---|---|---|---|---|---|---|---|---|
| COH | p: 6.8e-9 | p: 3.9e-6 | p: 8.0e-3 | p: 1.2e-8 | p: 0.037 | p: 6.0e-7 | p: 4.2e-8 | p: 6.5e-9 |
| PLV | | p: 5.4e-9 | p: 1.6e-5 | p: 6.7e-11 | p: 2.2e-4 | p: 1.1e-9 | p: 1.3e-10 | p: 3.3e-11 |
| CIPLV | | | p: 3.9e-3 | p: 1.4e-3 | p: 3.5e-4 | p: 0.020 | p: 2.7e-4 | p: 2.8e-3 |
| ECSO | | | | p: 2.8e-5 | p: 0.73 | p: 2.3e-4 | p: 5.0e-5 | p: 4.5e-5 |
| ECPWO | | | | | p: 3.5e-5 | p: 0.035 | p: 0.092 | p: 0.48 |
| IMCOH | | | | | | p: 5.0e-5 | p: 2.5e-5 | p: 3.8e-5 |
| WPLI | | | | | | | p: 0.028 | p: 0.084 |
| PLI | | | | | | | | p: 0.40 |

**Self-Stability Percentile**

| | PLV | CIPLV | ECSO | ECPWO | IMCOH | WPLI | PLI | PSI |
|---|---|---|---|---|---|---|---|---|
| COH | p: 5.7e-11 | p: 4.7e-6 | p: 0.77 | p: 1.8e-11 | p: 8.5e-8 | p: 1.8e-11 | p: 5.6e-12 | p: 1.9e-16 |
| PLV | | p: 5.1e-10 | p: 2.9e-3 | p: 8.4e-14 | p: 3.8e-10 | p: 2.8e-13 | p: 7.5e-14 | p: 1.3e-18 |
| CIPLV | | | p: 2.1e-5 | p: 1.1e-7 | p: 9.8e-6 | p: 1.0e-11 | p: 1.8e-12 | p: 2.3e-14 |
| ECSO | | | | p: 3.2e-11 | p: 1.5e-8 | p: 1.8e-11 | p: 4.1e-12 | p: 6.8e-18 |
| ECPWO | | | | | p: 0.10 | p: 0.21 | p: 0.13 | p: 1.7e-4 |
| IMCOH | | | | | | p: 2.5e-4 | p: 2.5e-5 | p: 5.8e-7 |
| WPLI | | | | | | | p: 0.34 | p: 8.2e-4 |
| PLI | | | | | | | | p: 1.7e-3 |

**Supplemental Figure 3. Statistical comparisons for beta band (13-30 Hz) connectivity between self-stability and identifiability measures, as presented in Figure 5 and Supplemental Figure 2.** Between each FC measure, a paired t-test was used to assess the statistical difference between participants' scores. Yellow shading = $p < 0.05$ Bonferroni corrected; green shading = $p < 0.05$ uncorrected. *COH: coherence; PLV: phase locking value; CIPLV: corrected imaginary phase locking value; ECSO: envelope correlation with symmetric orthogonalization; ECPWO: envelope correlation with pairwise orthogonalization; IMCOH: imaginary coherence; WPLI: weighted phase lag index; PLI: phase lag index; PSI: phase slope index.*

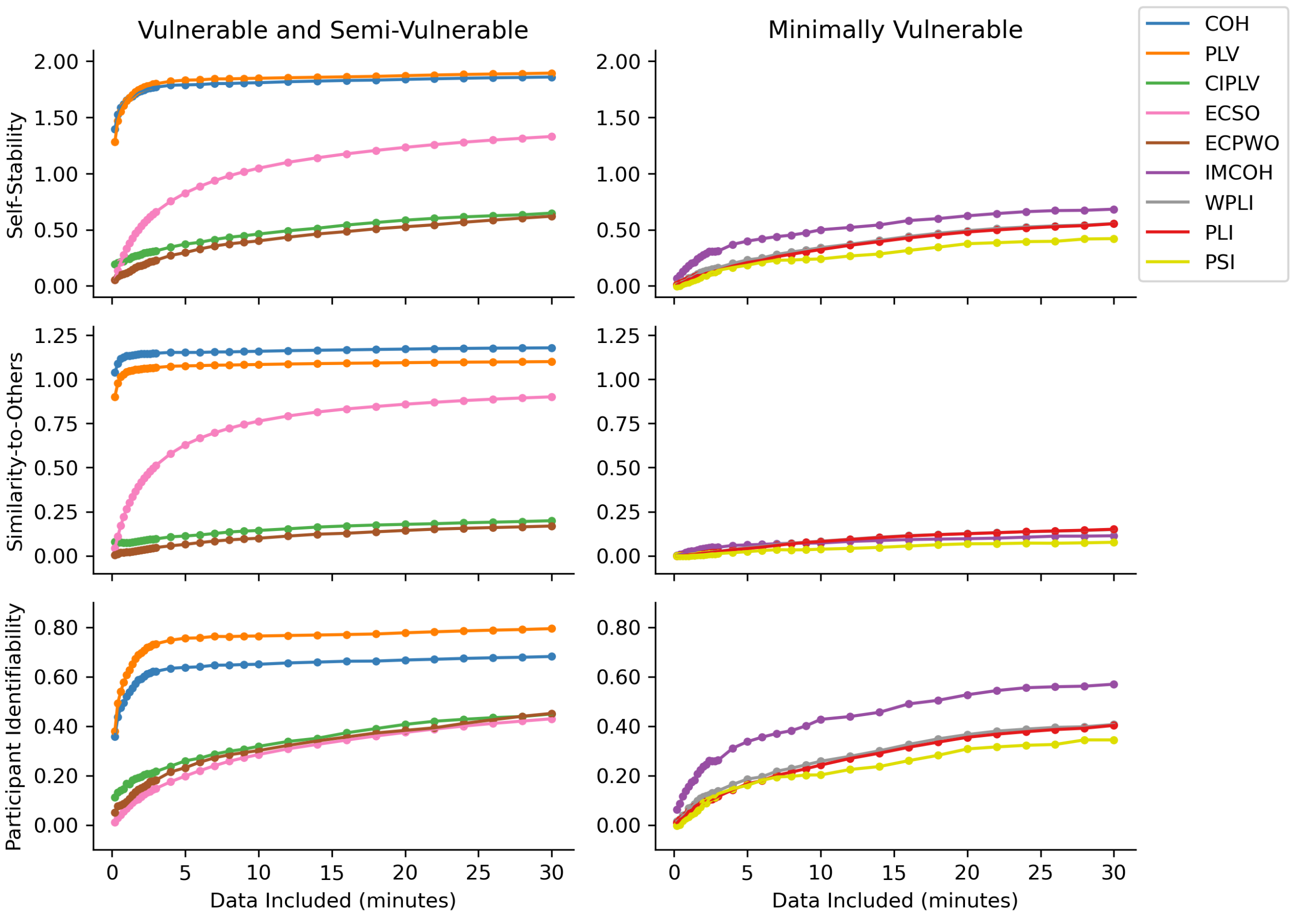


**Figure 6. Effect of different recording lengths on self-stability and identifiability, across FC measures, for beta band (13-30 Hz) connectivity.** Points shown are across-participant averages. For each participant, the recordings from their first two sessions and from their last two sessions were concatenated, giving two sets of data per participant. Data included refers to the total amount of data in each set, e.g., "12 minutes included" would refer to using the first 2 minutes available from each recording, 6 recordings in the first two sessions and 6 in the last two sessions. From top to bottom, subplots represent: **Mean self-stability**, the average Fisher-z correlation between connectomes of the same participant, averaged across participants. **Mean similarity-to-others**, the average Fisher-z correlation between a participant's connectomes and connectomes from all other participants, averaged across participants. **Mean participant identifiability**, the difference between self-stability and mean similarity-to-others, averaged across participants. *COH: coherence; PLV: phase locking value; CIPLV: corrected imaginary phase locking value; ECSO: envelope correlation with symmetric orthogonalization; ECPWO: envelope correlation with pairwise orthogonalization; IMCOH: imaginary coherence; WPLI: weighted phase lag index; PLI: phase lag index; PSI: phase slope index.*

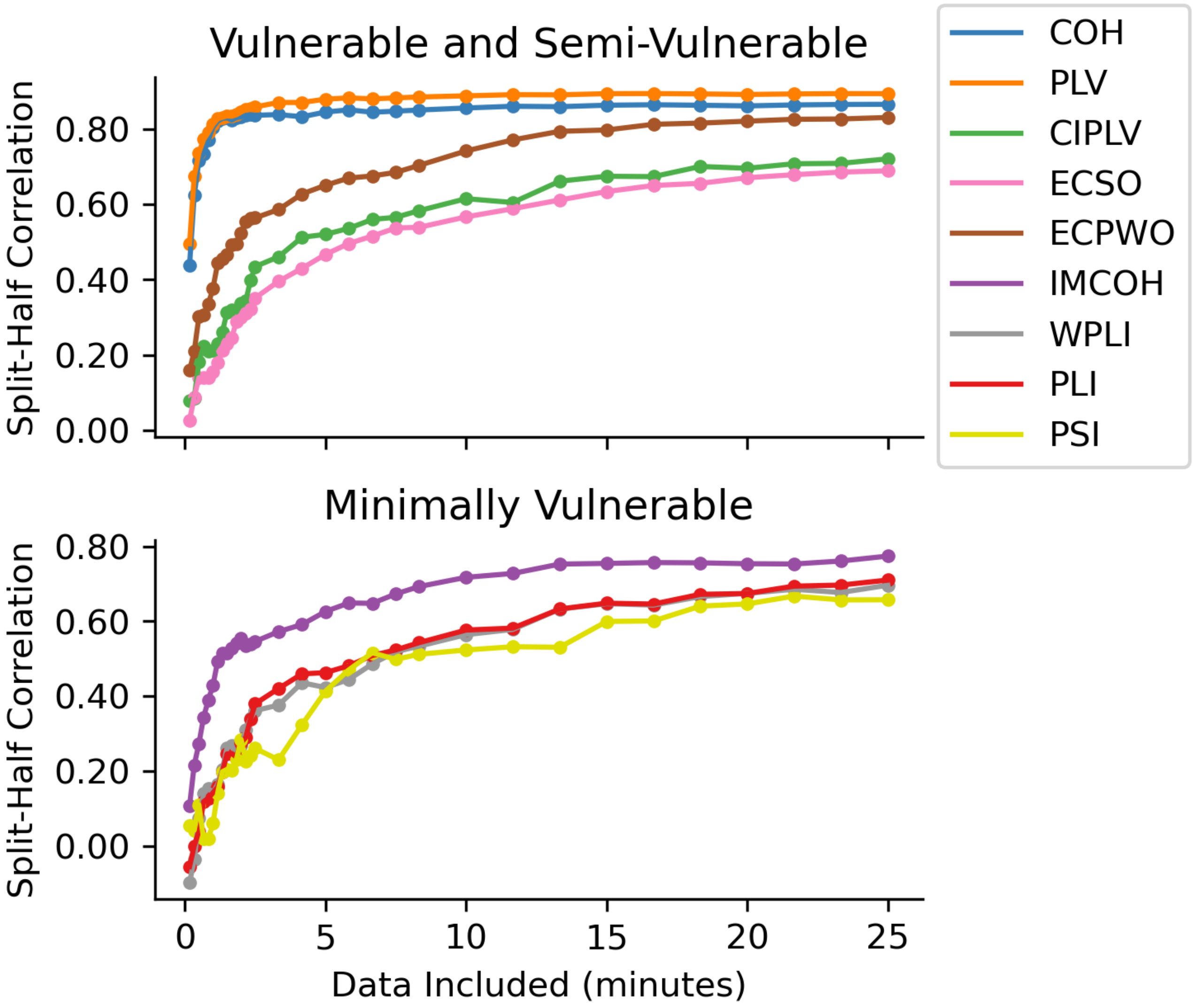


**Figure 7. Effect of different recording lengths on the stability of age group effects across recording sessions for beta band (13-30 Hz) connectivity.** For each participant, the recordings from their first two sessions and from their last two sessions were concatenated, giving two sets of data per participant, from which connectomes were generated. Across parent-child pairs, using each participant's first set of data, paired t-tests were used to calculate t values of age group effects for each connectivity edge. This was repeated for the second set of data, and the two sets of t values were then correlated, to assess the stability of measured age group effects. Data included refers to the total amount of data in each set, e.g., "10 minutes included" would refer to using the first 2 minutes available from each recording, 5 recordings in the first two sessions and 5 in the last two sessions. *COH: coherence; PLV: phase locking value; CIPLV: corrected imaginary phase locking value; ECSO: envelope correlation with symmetric orthogonalization; ECPWO: envelope correlation with pairwise orthogonalization; IMCOH: imaginary coherence; WPLI: weighted phase lag index; PLI: phase lag index; PSI: phase slope index.*
